# A Similarity Matrix for Preserving Haplotype Diversity Amongst Parents in Genomic Selection

**DOI:** 10.1111/jbg.12930

**Published:** 2025-03-04

**Authors:** Abdulraheem A. Musa, Norbert Reinsch

**Affiliations:** ^1^ Research Institute for Farm Animal Biology (FBN) Dummerstorf Germany

**Keywords:** breeding value, genetic gain, genetic variance, genomic selection, haplotype similarity, index selection, mendelian sampling, optimal contribution, within‐family variance

## Abstract

In genomic selection, balancing genetic gain with the preservation of genetic diversity is a critical challenge, requiring innovative approaches to parent selection. Traditional methods risk losing valuable genetic diversity by not fully accounting for the complex patterns of haplotype distribution. To address this, we developed a novel haplotype similarity measure that estimates the genetic similarity amongst offspring from parent pairs by analysing segregating marker patterns and the covariance of additive genetic effects between potential parental gametes. This measure is encapsulated in a novel similarity matrix that quantifies parental genetic relationships and their Mendelian sampling variance, facilitating the selection of parents with diverse haplotypes to maintain genetic diversity. Our method was evaluated through simulation studies and empirical data analysis, indicating that the similarity matrix can help preserve haplotype diversity and potentially improve long‐term genetic gains compared to traditional selection methods. These results suggest that the similarity matrix could contribute to more efficient and sustainable genomic selection programs, although further research is necessary to fully understand its impact.

## 1 Introduction

Genomic selection (GS) has revolutionised breeding by increasing the accuracy of estimated breeding values (BVs) of young candidates and shortening the generation intervals, thereby accelerating genetic gain (Meuwissen et al. 2001; Heffner et al. 2009; Hickey et al. 2017). Conventional GS often relies on truncation selection (TS), where parents are selected solely based on their genomic estimated breeding values (GEBVs). The application of TS, however, varies significantly across different species and breeding contexts. GEBVs can be derived in various ways: in marker‐based BLUP (Best Linear Unbiased Prediction), GEBVs are calculated by summing the estimated additive effects of all alleles in an individual's genotype to maximise the average genetic value of the next generation. Alternatively, genomic BLUP (G‐BLUP) directly computes GEBVs using a genomic relationship matrix, bypassing the explicit calculation of individual marker effects. Importantly, both methods are statistically equivalent under Henderson's mixed model equations, allowing for conversions between marker effects and GEBVs as needed (Henderson 1984). However, the continued use of this approach has significant drawbacks, including the promotion of inbreeding and the loss of genetic diversity, jeopardising long‐term genetic gains (Schaeffer 2006; Liu et al. 2015; Rutkoski et al. 2015; Lin et al. 2016).

To mitigate these drawbacks, two strategic directions have emerged. The first emphasises inbreeding control, as seen with optimal contribution selection (OCS), which employs coancestry constraints to balance genetic gains with the loss of genetic diversity (Meuwissen and Sonesson 1998; Sonesson et al. 2012; Woolliams et al. 2015). While OCS effectively manages genetic diversity and inbreeding, its dependency on accurate estimates of coancestry and genetic relationships can pose challenges in populations where pedigree or genomic information is incomplete or error‐prone. The second strategy focuses on preserving haplotype diversity, using chromosome segmentation methods like genotype building and optimal haploid value selection (Kemper et al. 2012; Daetwyler et al. 2015). These methods select parents based on haplotype blocks or segmental GEBVs but may not fully account for quantitative trait loci (QTLs) or linkage disequilibrium (LD) effects (Lehermeier et al. 2017; Müller et al. 2018). Kemper et al. (2012) found that OCS outperforms genotype building in maintaining genetic diversity and controlling inbreeding, especially in low coancestry scenarios, by enabling more flexible selection of genetic segments. Conversely, genotype‐building strategies may achieve higher maximum response rates when coancestry constraints are relaxed, as they focus on specific high‐value segments. Additionally, look‐ahead selection, distinct from optimal population value selection, aims to maximise the expected GEBV of the best progeny in the terminal generation, a process that is notably computationally intensive (Goiffon et al. 2017; Moeinizade et al. 2019).

Steering away from chromosome segmentation methods, recently introduced genomic selection criteria are based on an index that combines the expected GEBVs of future offspring with Mendelian sampling variance (MSV), a measure of the within‐family genetic variability. This MSV, inferred from parental alleles and additive marker effects, is predicted either through the simulation of the segregation of parental haplotypes into gametes (Cole and VanRaden 2011; Bernardo 2014; Segelke et al. 2014; Mohammadi et al. 2015) or by applying an analytical formula (Bonk et al. 2016; Santos et al. 2019). These indices, including the ‘usefulness criterion’ and the ‘expected maximum haploid breeding value’, are relevant in plant breeding with doubled haploid lines (Schnell and Utz 1976; Lehermeier et al. 2017; Müller et al. 2018). Additionally, variants of these indices have been applied to diploid animals, indicating the potential for substantial long‐term genetic gains (Bijma et al. 2020). However, despite their promise of enhancing long‐term genetic gains, these indices are limited by the computationally intensive calculations of MSV, which can hinder large‐scale applications. Moreover, they lack intrinsic mechanisms inherent in the chromosome segmentation methods to preserve haplotype diversity. This limitation can lead to the inadvertent loss of favourable haplotypes, particularly those with small effects, countering breeders' objectives of preserving a wide spectrum of haplotypes. Such diversity is essential to minimise the loss of genetic variability and foster the emergence of new favourable haplotypes through recombination—a process vital for the continuous improvement and adaptation of species.

To tackle these computational challenges and the vital need to preserve genetic diversity, this study introduces a novel approach that utilises a computationally efficient representation of an analytical method inspired by Bonk et al. (2016) to predict the MSV of gametes. Our method employs a covariance matrix that captures expected within‐family LD, enabling the derivation of a metric for quantifying pairwise haplotype similarity between parents, initially focused on a single chromosome. This matrix method offers a nuanced perspective on MSVs and parent similarities, expanding to include multiple chromosomes, traits, and zygotes. Building upon the foundational genomic selection methods, such as chromosome segmentation by Kemper et al. (2012), we introduce an innovative approach that streamlines the process. Our method sidesteps the need for segmental GEBVs, instead directly quantifying pairwise haplotype similarity. Additionally, while sharing foundational principles with OCS in managing genetic diversity, our approach utilises a similarity matrix based on MSV instead of a coancestry matrix. We advocate this matrix as a new pathway for optimising genetic gains while preserving haplotype diversity within breeding programs, addressing a significant shortfall in current genomic selection criteria based on the index. Finally, we demonstrate the potential applications of this similarity matrix in preserving haplotype diversity through simulation.

## 2 Materials and Methods

In this section, we first review the method of Bonk et al. (2016) for calculating MSV and then present an equivalent but computationally faster representation. This representation will also be useful in defining our new similarity measure and its computation with large numbers of markers and as many individuals as needed for potential applications inoptimised selection or mating decisions.

### 2.1 Computational Advances in MSV Prediction

Here, we revisit the analytical formula proposed by Bonk et al. (2016) for estimating the MSV to establish the basis for subsequent computational improvements. Their formula presented a significant enhancement over prior simulation‐based estimations such as those by Segelke et al. (2014), and Mohammadi et al. (2015). While these prior methods used the same information, such as recombination rates, marker effects, and phased genotypes of parents, they were prone to the stochastic errors characteristic of simulation modelling, such as Monte Carlo. The MSV is inherently linked to the breeding value of individual parents, as it captures the variance in the genetic contribution that can be passed on to offspring. The breeding value, denoted as $b_{i}$ for parent i, is the inner product of a row vector $c_{i}^{\prime }$, and a column vector of the population's additive marker effects (or allele substitution effects) m, thus:

(1) $$b_{i}=c_{i}^{\prime }m$$

Here, $c_{i}^{\prime }$ consists of genotype indicators for biallelic markers, where 1, 0, and −1 correspond to homozygous reference, heterozygous, and homozygous alternate genotypes, respectively.

The MSV of the BV of gametes produced by a potential parent i is subsequently computed as:

(2) $$\mathrm{var}\left(b_{i}\right)=m^{\prime }R_{i}m$$

In this formula, $R_{i}$ represents a parent‐specific covariance matrix for additive genotype indicators in $c_{i}^{\prime }$, which is derived from the parent's recombination rates and linkage phases of markers. The matrix $R_{i}$ mirrors the within‐family LD of markers, with its structure being a block‐diagonal matrix, where each chromosome forms a block. Using a suitable mapping function such as that proposed by Haldane (1919), the covariance elements $\rho_{\mathrm{kl}}$ of $R_{i}$ can be expressed in terms of genetic distances, using the following expression:

(3) $$\rho_{\mathrm{kl}}=\pm \frac{\exp\left(-2d_{\mathrm{kl}}\right)}{4}$$

The sign of $\rho_{\mathrm{kl}}$ is contingent on the linkage phase between markers k and l, and $d_{\mathrm{kl}}$ represents the genetic distance between these markers in Morgans. The diagonal elements have a value of 0.25 when $d_{\mathrm{kl}}=0$, while off‐diagonal elements corresponding to markers on different chromosomes are zero, indicative of $d_{\mathrm{kl}}=\infty$. Rows and columns in $R_{i}$ pertaining to homozygous markers are set to zero since they do not contribute to MSV.

To estimate MSV in zygotes resulting from the mating of parents i and j, the BVs $b_{\mathrm{ij}}$ are the sum of the independent gamete contributions from each parent. This summation implies that the MSV of the zygote can be partitioned into the variances attributable to each parent, as indicated by:

(4) $$\mathrm{var}\left(b_{\mathrm{ij}}\right)=m^{\prime }R_{i}m+m^{\prime }R_{j}m$$

#### 2.1.1 Computational Improvements for MSV Prediction

We propose an equivalent but computationally faster representation of the analytical formula for predicting MSV. This representation simplifies the derivation of an expected within‐family LD matrix R, akin to $R_{i}$, but with positive elements and no nullification of elements corresponding to homozygous markers. Additionally, we modify the additive marker effects vector m into a parent‐specific additive marker effects vector $m_{i}$, which integrates the linkage phases of markers. The parent‐specific vector $m_{i}$ is defined such that for each marker k, a phase‐indicator variable $\delta_{\mathrm{ik}}$ is set to 1 if the reference allele is present on the first haplotype of the individual; otherwise, it is set to −1. For any homozygous genotype, $\delta_{\mathrm{ik}}$ is set to 0. Thus, the additive marker effects $m_{k}$ are thus transformed into $m_{\mathrm{ik}}=\delta_{\mathrm{ik}}m_{k}$, preserving the sign of m in Equation (2) if the reference allele is on the first haplotype; otherwise, the sign is reversed if the genotype is heterozygous. The resulting equation for the MSV of BVs of gametes for parent i is then:

(5) $$\mathrm{var}\left(b_{i}\right)=m_{i}^{\prime }{\mathrm{Rm}}_{i}$$

In this representation, matrix R is established only once and is universally applicable to all parental marker pairs, eliminating the need for repetitive adjustments as in $R_{i}$. Furthermore, setting up the $m_{i}$ vectors for individual markers is significantly simpler than the previous method, which required exhaustive pairwise marker exploration to set up $R_{i}$. This streamlined representation significantly accelerates MSV computation compared to previously presented formulas (Bonk et al. 2016; Santos et al. 2019; De Abreu Santos et al. 2020). Additionally, this novel representation plays a pivotal role in deriving our similarity measure.

Furthermore, it is worth noting that swapping haplotypes within the vector $m_{i}$ results in an identical vector with opposite signs $-m_{i}$. However, the resulting MSV remains unaffected, regardless of the chosen order of haplotype. See Appendices A and B for an illustration and proof of the equivalence of Equations (2) and (5).

#### 2.1.2 Extension to Multiple Traits, Chromosomes, and Aggregate Genotype

In practice, breeders often select parents based on multiple traits, requiring a methodology that accounts for both the MSVs and the covariances amongst these traits. To address this, we extend the single‐trait MSV $m_{i}^{\prime }{\mathrm{Rm}}_{i}$ to the case of multiple traits $b_{i}$, which is denoted as $\mathrm{var}\left(b_{i}\right)$

(6) $$\mathrm{var}\left(b_{i}\right)=M_{i}RM_{i}^{\prime }$$

Here, each row of the matrix $M_{i}$ represents a vector $m_{\mathrm{it}}^{\prime }$ of additive marker effects for each trait t. Our methodology can be readily extended to encompass multiple chromosomes by summing up the contributions from each chromosome c:

(7) $$\mathrm{var}\left(b_{i}\right)=\sum_{c}M_{i}^{c}R^{c}{\left(M_{i}^{c}\right)}^{\prime }=V_{i}$$

Here, $R^{c}$ is the diagonal block of R pertaining to chromosome c, and each row of the matrix $M_{i}^{c}$ represents a vector $m_{\mathrm{it}}^{\prime }$ of additive marker effects for each trait t on the same chromosome. The diagonal elements of the Mendelian covariance matrix $V_{i}$ represent the MSVs of all traits, while the off‐diagonal elements capture the Mendelian sampling covariances (MSCs) between traits, as originally elucidated by Bonk et al. (2016). Finally, by assuming a known vector of index weight a for all traits, the MSV for the aggregate genotype becomes:

(8) $$\mathrm{var}\left(a^{\prime }b_{i}\right)=a^{\prime }\cdot V_{i}\cdot a$$

#### 2.1.3 Extension to Zygotes

We propose computing the MSV of progeny produced by parents i and j in the same way as described in Equation (4):

(9) $$\mathrm{var}\left(b_{\mathrm{ij}}\right)=m_{i}^{\prime }{\mathrm{Rm}}_{i}+m_{j}^{\prime }{\mathrm{Rm}}_{j}$$

again, where R is the covariance matrix reflecting the expected within‐family LD of markers, and $m_{i}$ ($m_{j}$) represents the parent‐specific additive marker effects vector for parent i (j).

For multiple traits, the MSV for zygotes from parents i and j is computed using:

(10) $$\mathrm{var}\left(b_{\mathrm{ij}}\right)=M_{i}RM_{i}^{\prime }+M_{j}RM_{j}^{\prime }$$

In this equation, each row $m_{\mathrm{it}}^{\prime }$ and $m_{\mathrm{jt}}^{\prime }$ of $M_{i}$ and $M_{j}$, respectively, represents vectors of the marker effects for trait t of parents i and j. Extending this framework to multiple chromosomes, we sum the contributions from each chromosome c to obtain the MSV for zygotes produced by parents i and j:

(11) $$\mathrm{var}\left(b_{\mathrm{ij}}\right)=\sum_{c}\left[M_{i}^{c}R^{c}{\left(M_{i}^{c}\right)}^{\prime }+M_{j}^{c}R^{c}{\left(M_{j}^{c}\right)}^{\prime }\right]=V_{\mathrm{ij}}$$

Here, each row of the matrix $M_{i}^{c}\left(M_{j}^{c}\right)$ represents a vector $m_{\mathrm{it}}^{\prime }\left(m_{\mathrm{jt}}^{\prime }\right)$ of additive marker effects for each trait t on the same chromosome. This matrix $V_{\mathrm{ij}}$ is the Mendelian covariance matrix of zygotes, with diagonal elements representing the MSVs of individual traits and off‐diagonal elements representing the MSCs between traits. For the aggregate genotype considering all traits, we calculate the MSV for zygotes produced by parents i and j as:

(12) $$\mathrm{var}\left(a^{\prime }b_{\mathrm{ij}}\right)=a^{\prime }\cdot V_{\mathrm{ij}}\cdot a$$

This equation quantifies the MSV for the aggregate BVs of zygotes, accounting for multiple traits and their covariances.

### 2.2 Similarities Between Haplotypes of Parents

#### 2.2.1 Conceptualising Pairwise Haplotype Similarity

To derive our similarity measure, we consider two parents with a single chromosome and all the gametes they produce. We choose an arbitrary order of haplotypes from both parents and assign a segregation pattern to each possible gamete. To illustrate our approach, we use three biallelic markers, creating eight distinct segregation patterns as depicted in Table 1.

**TABLE 1:** Segregation patterns (sequence of alleles from the first and second parental haplotypes), probabilities of occurrence, and breeding values of pairs of gametes produced by the first and second parents with matching recombination patterns.

Segregation pattern/Gametes produced	Probability	Breeding value	
		First parent	Second parent
A−A−A	$\frac{1}{2}\cdot \left(1-\theta_{1,2}\right)\cdot \left(1-\theta_{2,3}\right)$	$\frac{1}{2}\left(m_{1}+m_{2}+m_{3}\right)=0.61$	$\frac{1}{2}\left(m_{1}-m_{2}+m_{3}\right)=0.41$
A−A−B	$\frac{1}{2}\cdot \left(1-\theta_{1,2}\right)\cdot \theta_{2,3}$	$\frac{1}{2}\left(m_{1}+m_{2}-m_{3}\right)=0.59$	$\frac{1}{2}\left(m_{1}-m_{2}-m_{3}\right)=0.39$
A−B−A	$\frac{1}{2}\cdot \theta_{1,2}\cdot \theta_{2,3}$	$\frac{1}{2}\left(m_{1}-m_{2}+m_{3}\right)=0.41$	$\frac{1}{2}\left(m_{1}+m_{2}+m_{3}\right)=0.61$
A−B−B	$\frac{1}{2}\cdot \theta_{1,2}\cdot \left(1-\theta_{2,3}\right)$	$\frac{1}{2}\left(m_{1}-m_{2}-m_{3}\right)=0.39$	$\frac{1}{2}\left(m_{1}+m_{2}-m_{3}\right)=0.59$
B−A−A	$\frac{1}{2}\cdot \theta_{1,2}\cdot \left(1-\theta_{2,3}\right)$	$\frac{1}{2}\left(-m_{1}+m_{2}+m_{3}\right)=-0.39$	$\frac{1}{2}\left(-m_{1}-m_{2}+m_{3}\right)=-0.59$
B−A−B	$\frac{1}{2}\cdot \theta_{1,2}\cdot \theta_{2,3}$	$\frac{1}{2}\left(-m_{1}+m_{2}-m_{3}\right)=-0.41$	$\frac{1}{2}\left(-m_{1}-m_{2}-m_{3}\right)=-0.61$
B−B−A	$\frac{1}{2}\cdot \left(1-\theta_{1,2}\right)\cdot \theta_{2,3}$	$\frac{1}{2}\left(-m_{1}-m_{2}+m_{3}\right)=-0.59$	$\frac{1}{2}\left(-m_{1}+m_{2}+m_{3}\right)=-0.39$
B−B−B	$\frac{1}{2}\cdot \left(1-\theta_{1,2}\right)\cdot \left(1-\theta_{2,3}\right)$	$\frac{1}{2}\left(-m_{1}-m_{2}-m_{3}\right)=-0.61$	$\frac{1}{2}\left(-m_{1}+m_{2}-m_{3}\right)=-0.41$

*Note: A* represents the alleles on the first haplotype, and *B* represents the alleles on the second haplotype. The first parent has ordered haplotypes of 1−1−1/2−2−2, while the second parent has ordered haplotypes of 1−2−1/2−1−2. Recombination rates between adjacent markers are assumed to be $\theta_{1,2}=.1$ and $\theta_{2,3}=.2$, and vector of population additive marker effects is assumed to be $m^{\prime }=\left[1\;.2\;.02\right]$. Pattern A−A−B, for example, has alleles 1−1−2 in the first parent and 1−2−2 in the second parent. Note that breeding value represents the value transmitted to the offspring.

For example, consider the pattern A−B−A. This pattern implies that alleles from the first parental haplotype (A) are transmitted at the first and third loci, while an allele from the second parental haplotype (B) is transmitted at the second locus. Recombination rates between adjacent markers, assumed to be $\theta_{1,2}=.1$ and $\theta_{2,3}=.2$, influence the probabilities associated with these segregation patterns, thus determining the probabilities of gametes with a particular segregation pattern. Importantly, while there exists a matching gamete from the second parent with an identical segregation pattern and an equivalent probability of occurrence for each gamete produced by the first parent, these events occur independently. This underscores that the matching patterns do not arise simultaneously. This distinction is crucial in appreciating the independent formation of gametes in each parent, governed by unique recombination events. While segregation patterns of gametes from both parents are identical, their additive values differ based on the alleles (1 or 2) present on the first (A) and second (B) haplotype of each parent, as detailed in Table 1. Specifically for the pattern A−B−A, the genetic value for the first parent, with haplotypes ordered as 1−1−1/2−2−2, is calculated as $\frac{1}{2}\left(m_{1}-m_{2}+m_{3}\right)$, resulting in a value of 0.41, given marker effects, $m_{1}=1$, $m_{2}=0.2$, and $m_{3}=0.02$. Similarly, for the second parent with haplotypes ordered as 1−2−1/2−1−2, the breeding value is computed as $\frac{1}{2}\left(m_{1}+m_{2}+m_{3}\right)$, yielding a value of 0.61.

#### 2.2.2 Estimating MSV and Covariance of Gametes

The additive value assigned to any segregation pattern depends on the haplotype order. As mentioned earlier, the MSV for each parent does not change if haplotypes are swapped and, therefore, can unequivocally be derived from the probability distribution of segregation patterns.

Given the segregation patterns and their associated probabilities, we compute the MSV of the BVs $b_{1}$ from the gametes produced by the first parent in the example is calculated as follows:

$$\begin{array}{cc} E\left(b_{1}^{2}\right)\; & =\sum_{w}p_{w}b_{1}^{w}=\frac{1}{2}\cdot 0.9\cdot 0.8\cdot 0.61^{2}\\ & +\ldots +\frac{1}{2}\cdot 0.9\cdot 0.8\cdot {\left(-0.61\right)}^{2}=0.3461 \end{array}$$

$$\begin{array}{cc} E\left(b_{1}\right)\; & =\sum_{w}p_{w}b_{1}^{w}=\frac{1}{2}\cdot 0.9\cdot 0.8\cdot 0.61\\ & +\ldots +\frac{1}{2}\cdot 0.9\cdot 0.8\cdot \left(-0.61\right)=0 \end{array}$$

$$\mathrm{var}\left(b_{1}\right)=E\left(b_{1}^{2}\right)-{\left(E\left[b1\right]\right)}^{2}=0.3461-0=0.3461$$

Here, $p_{w}$ denotes the probability of the segregation pattern w, and $b_{1}^{w}$ represents the additive value of the gamete transmitted from the first parent. The MSV for the second parent is determined similarly, yielding:

$$\mathrm{var}\left(b_{2}\right)=E\left(b_{2}^{2}\right)-{\left(E\left[b2\right]\right)}^{2}=0.1837$$

We then extend this approach to compute the covariance between the BVs of matching gametes from both parents. This covariance is conditional on the chosen order of haplotypes. With $E\left(b_{1}\right)=E\left(b_{2}\right)=0$, this covariance in our example is

$$E\left(b_{1},b_{2}\right)=\sum_{w}p_{w}b_{1}^{w}b_{2}^{w}=0.2449=\mathrm{cov}\left(b_{1},b_{2}\right)$$

We emphasise that expectations are based on the univariate conditional distribution of segregation patterns rather than the bivariate joint distribution of gametes from two parents. The latter would result in a zero covariance because of the independence of the Mendelian sampling processes in different individuals.

When dealing with hundreds to thousands of markers, enumerating all segregation patterns and their probabilities becomes impractical. However, the adoption of matrix expressions, as mentioned by Bonk et al. (2016), simplifies the computation of variances and covariances, even with many markers.

#### 2.2.3 Equivalent Matrix Expressions

Similarities and MSV can be conceptualised as a dot product, effectively defining the cosine of the angle between family‐specific marker vectors. For unlinked markers, where the linkage phase is not a consideration (assuming R=I/4, where I is an identity matrix), we construct a specialised vector ${\tilde{m}}_{i}$. This vector ${\tilde{m}}_{i}$ is formulated by setting all homozygous loci in the original marker effects vector m to zero, retaining only the values at heterozygous loci. Thus, the MSV for a parent i is expressed as the dot product ${\tilde{m}}_{i}^{\prime }{\tilde{m}}_{i}$, and this value is scaled by 1/4 to adjust for the variance contribution from each heterozygous locus. For two parents i and j, the similarity is expressed as ${\tilde{m}}_{i}^{\prime }{\tilde{m}}_{j}=\left\|{\tilde{m^{\prime }}}_{i}\right\|\left\|{\tilde{m}}_{j}\right\|\cos\left(\alpha \right)$, where $\cos\left(\alpha \right)$ is always positive, representing the sum of squared marker effects for all markers that are heterozygous in both parents.

For linked markers, utilising the Cholesky decomposition ($R=LL^{\prime }$) enables the expression of the MSV of parent i as the dot product ${\left(L\cdot m_{i}\right)}^{\prime }\cdot \left(L\cdot m_{i}\right)$. Similarly, the similarity between parents i and j, is given by ${\left(L\cdot m_{i}\right)}^{\prime }\cdot \left(L\cdot m_{j}\right)=\left\|{\left(L\cdot m_{i}\right)}^{\prime }\right\|\left\|\left(L\cdot m_{j}\right)\right\|\cos\left(\alpha \right)$. In this context, $\cos\left(\alpha \right)$ remains positive for all cases where $\alpha =\alpha^{a}$ is the acute angle $0\leq \alpha^{a}<\pi /2$. Conversely, $\cos\left(\alpha \right)$ will be negative if $\alpha =\alpha^{s}$ is the supplementary angle of $\alpha^{a}$ since $\alpha^{a}+\alpha^{s}=\pi$. Since $\cos\left(\pi -\alpha \right)=-\cos\left(\alpha \right)$, the convention is to consistently opt for the smaller angle α, rather than the supplementary angle π−α. This convention ensures that the cosine of the angle, which dictates the sign and magnitude of the similarity, remains consistent and meaningful.

This convention becomes particularly insightful when considering a pair of monozygous twins. Without this convention, their similarity would be either 1 or −1 for a single chromosome. With multiple chromosomes, the result would be an unpredictable sum of positive and negative contributions from different chromosomes, depending on the combination of haplotype orders for all chromosomes, with a maximum value of one. Employing absolute values in the similarity calculation ensures that the measure remains consistent and accurately reflects the true biological relationship, not influenced by the arbitrary order of haplotypes.

Furthermore, in practical applications, particularly in large genomic datasets, a unique similarity measure $s_{i,j}$ valid for any possible order of parental haplotypes of parents i and j can be defined as a bilinear form, denoted as:

(13) $$s_{i,j}=\left|m_{i}^{\prime }{\mathrm{Rm}}_{j}\right|$$

again, where R is as defined in Equation (5), and $m_{i}$ ($m_{j}$) represents the parent‐specific additive marker effects vector for parent i (j).

The bilinear form provides a computationally efficient method to calculate similarities without requiring matrix decomposition. This single bilinear operation significantly improves computational efficiency. For specific numerical examples pertaining to the parents in our example (Table 1), please consult (Data S1: A.1–A.3).

#### 2.2.4 Extension to Chromosomes, Multiple Traits, and Aggregate Genotype

For a genome with several chromosomes, the similarity is calculated as the sum of absolute contributions from all chromosomes

(14) $$s_{i,j}=\sum_{c}\left|{\left(m_{i}^{c}\right)}^{\prime }R^{c}m_{j}^{c}\right|$$

where $R^{c}$ is the diagonal block of R pertaining to chromosome c, and $m_{i}^{c}$($m_{j}^{c}$) represents the parent‐specific additive marker effects vector for parent i (j) on the same chromosome. An individual's similarity to itself (i.e., i=j) equals that individual's MSV, which is always positive and independent of haplotype order. Therefore, it is possible to assemble a trait‐specific similarity matrix S with MSVs for each parent on the diagonal and pairwise similarities $s_{i,j}$ as off‐diagonal elements for any set of individuals.

When considering multiple traits, there is also a similarity between potential parents i and j with respect to the aggregate genotype. Each element $s_{\mathrm{ij}}$ of the respective similarity matrix is given by the following equation:

$$s_{i,j}=\sum_{c}\left|a^{\prime }\cdot \left(M_{i}^{c}R_{c}{\left(M_{j}^{c}\right)}^{\prime }\right)\cdot a\right|$$

#### 2.2.5 Extension to Zygotes

A common breeding interest is determining the optimal parents for mating and the number of mates to assign to each parent, which can be determined using a gametic similarity matrix. However, in certain scenarios like multiple ovulation and embryo transfer, the focus extends to determining the optimal parent pairs and the ideal number of offspring they should produce.

Hence, it becomes valuable to quantify the genetic similarity between the zygotes produced by these specific parent pairs. Similar to gametes, this measure can also be calculated from the probability distribution (see B.1 in Data S1 for the derivation). Furthermore, expressing this similarity using matrix notation offers a more efficient approach, particularly when dealing with numerous genetic markers. If $m_{i}$, $m_{j}$, $m_{u}$, and $m_{v}$ are the marker effect vectors of parents i, j, u, and v, then the similarity between the BVs of zygotes produced by parent pairs ij and uv in the case of a single chromosome is:

(15) $$s_{\mathrm{ij},\mathrm{uv}}=\left|m_{i}^{\prime }{\mathrm{Rm}}_{u}\right|+\left|m_{j}^{\prime }{\mathrm{Rm}}_{v}\right|$$

For multiple chromosomes, we sum over all chromosomes:

(16) $$s_{\mathrm{ij},\mathrm{uv}}=\sum_{c}\left(\left|{\left(m_{i}^{c}\right)}^{\prime }R^{c}m_{u}^{c}\right|+\left|{\left(m_{j}^{c}\right)}^{\prime }R^{c}m_{v}^{c}\right|\right)$$

Finally, these similarities $s_{\mathrm{ij},\mathrm{uv}}$ define the off‐diagonal elements of a similarity matrix S between pairs of parents (families) with respect to the makeup of the haplotypes in the zygotes of these families. The MSV of the respective family (which is equal to the similarity of a family with itself) represents the diagonal elements $s_{\mathrm{ij},\mathrm{ij}}$. Numerical examples of the derivation of MSV and the similarity between the zygotes of parent pairs ij and uv are also provided in (Data S1: B.1).

For two pairs of parents ij and uv, the similarity measure $s_{\mathrm{ij},\mathrm{uv}}$ for the aggregate genotype, which does not depend on the haplotype order of any of the four involved parents i, j, u, and v, becomes

$$s_{\mathrm{ij},\mathrm{uv}}=\sum_{c}\left[\left|a^{\prime }\cdot \left(M_{i}^{c}R^{c}{\left(M_{u}^{c}\right)}^{\prime }\right)\cdot a\right|+\left|a^{\prime }\cdot M_{j}^{c}R^{c}{\left(M_{v}^{c}\right)}^{\prime }\cdot a\right|\right]$$

Then, the MSVs of the aggregate genotype define the diagonal elements, and the similarities $s_{\mathrm{ij},\mathrm{uv}}$ define the off‐diagonal elements of the similarity matrix S between pairs of parents. See Appendix C for the extension of the similarity measure to monoecious species and the order of parents in dioecious organisms.

#### 2.2.6 Standardised Similarity

The similarity matrix S of either gametes or zygotes can be standardised by pre‐ and post‐multiplying with a diagonal matrix $D^{-1}$ with inverse Mendelian standard deviations as diagonal elements:

(17) $$K=D^{-1}\cdot S\cdot D^{-1}$$

where $D=\sqrt{\mathrm{diag}\left(S\right)}$. Thus, all diagonal elements of K are equal to 1, and all off‐diagonal elements are strictly non‐negative and lie within the range of $0\leq k_{\mathrm{ij}}\leq 1$.

### 2.3 Applications of Similarity Matrices in Genomic Selection

This section describes the methodology employed for utilising similarity matrices in Mendelian sampling‐based optimal contribution selection (MOCS). The entire process encompasses empirical data analysis and simulated data experiments.

#### 2.3.1 MOCS Scheme

The MOCS scheme aims to achieve optimal parent contributions $n_{t}$ in generation t by maximising the average expected genetic return ${\bar{r}}_{t+1}$ in the next generation, while adhering to a specified constraint on the average haplotype similarity ${\bar{Q}}_{t+1}$. The mathematical representation of the MOCS scheme is as follows:

(18) $$\begin{array}{l} \mathrm{maximize}\;{\bar{r}}_{t+1}=n_{t}^{\prime }r_{t}\;\text{with respect to}\;n_{t}\\ \text{with constraints}:\;{\bar{Q}}_{t+1}\leq n_{t}^{\prime }Q_{t}n_{t}/2\\ \;n_{t}^{\prime }v_{f}=1/2\\ \;n_{t}^{\prime }v_{m}=1/2\\ \;n_{t_{i}}^{\prime }\geq 0 \end{array}$$

Here, the vector $r_{t}$ represents the selection criterion, which may include BVs or indices combining BVs and MSV parents in generation t. $Q_{t}$ is a similarity matrix representing either S or K for gametes in generation t. Vectors $v_{f}$ and $v_{m}$ are indicator vectors (0/1) denoting females and males, respectively. This optimisation strategy can be extended to parent pairs by deriving $r_{t}$ and $Q_{t}$ for parent pairs and considering user‐specific constraints like maximum/minimum contributions of parent pairs.

While the theoretical similarity matrix S is positive semidefinite, practical scenarios may lead to indefiniteness due to rank deficiency or numerical inaccuracies. In such cases, the similarity matrix can be approximated to the nearest positive definite matrix. The linear optimisation problem with quadratic constraints is then solved using efficient algorithms to obtain the optimal parent contributions $n_{t}$ for any given dataset.

#### 2.3.2 Empirical Data

For the empirical validation of our study, we utilised a comprehensive dataset from Hampel et al. (2018), which consisted of five paternal half‐sib families of Holstein‐Friesian cows. This publicly accessible dataset encompasses 265 individuals, with each paternal half‐sib family varying in size, ranging from 32 to 106 individuals. It includes detailed pedigree information, genotypic data, and a physical map. The genetic map positions were derived by converting the physical map positions, expressed in megabase pairs (Mbp), to centimorgans (cM). The genotypic dataset was initially extensive, featuring 39,780 markers located on autosomes. However, the genotypes underwent phasing using *hsphase* version 2.0.2 (Ferdosi et al. 2014), which is well‐suited for datasets with complex or incomplete pedigrees. This phasing process, challenged by the limited pedigree depth inherent to half‐sib family data, resulted in many unknown phases, necessitating the refinement of the dataset to 10,304 markers after excluding those with unresolved phases to ensure the accuracy of our genetic analysis. The marker effects for this population were previously estimated in a study by Melzer et al. (2013).

We incorporated all 265 individuals in our analysis to generate similarity matrices, employing the gametic approach. To effectively visualise the haplotype diversity within this bovine population, these matrices were graphically represented using *corrplot* version 0.92 (Wei and Viliam 2021). Additionally, family‐wise clustering was performed to elucidate familial genetic structures, utilising *pheatmap* version 1.0.12 (Kolde 2019).

To interpret haplotype similarities between parents in our study, we conducted a linear regression analysis, examining the interplay between these similarities, common marker heterozygosity, and marker effect sizes. We calculated common heterozygosity by counting the number of heterozygous markers shared between two parents. Aggregated marker effects were assessed similarly, but with the addition of weighting by marker effects, resulting in the weighted sum of shared heterozygous marker effects. We also investigated the correlation between haplotype similarities, common marker heterozygosity, and marker effect sizes. Our analysis encompassed both dependent and independent marker scenarios to provide a thorough understanding of these relationships. For a detailed explanation of these scenarios, including the modelling of markers in our matrix expressions and their impact on the computed similarity calculations, refer to the section on ‘Equivalent Matrix Expressions’. The results of this multifaceted analysis were then visualised in three dimensions using the *plot3D* version 1.4.1 (Soetaert 2024), to offer valuable insights into the underlying dynamics.

#### 2.3.3 Simulated Data

We conducted experiments using simulated data to demonstrate the use of similarity matrices for preserving haplotype diversity. The simulated base population comprised 1000 cattle (500 males and 500 females) with 10 chromosomes, generated using *AlphaSimR* version 1.3.4 (Gaynor et al. 2021). We employed default genomic and demographic parameters for cattle as outlined in MacLeod et al. (2013), which include values for effective population size, LD, and marker allele frequencies. A sex‐averaged genetic map with an average chromosome length of 1 M was assumed. The genome included 10,000 single nucleotide polymorphisms (SNPs) and 2000 QTLs, with 1000 SNPs and 200 QTLs on each chromosome. Additive QTL effects were simulated from a normal distribution with a mean of 0 and a variance of 100. These effects were summed to derive true BVs for all individuals, with a trait heritability of 0.25 simulated by adding residual effects to the BVs.

Parent selection and mating were carried out over 50 generations, starting from the same base population, using various selection schemes (Table 2). These schemes combined selection criteria (BV and index) and rules (TS and MOCS).

**TABLE 2:** Overview of all selection schemes.

Truncation selection	MOCS			
	0.6	0.4	0.3	0.2
BV	BV_S_0.6_	BV_S_0.4_	BV_S_0.3_	BV_S_0.2_
	BV_K_0.6_	BV_K_0.4_	BV_K_0.3_	BV_K_0.2_
Index	Index_K_0.6_	Index_K_0.4_	Index_K_0.3_	Index_K_0.2_

*Note:* The indices 0.6, 0.4, 0.3, and 0.2 represent the 60th, 40th, 30th, and 20th percentiles of haplotype similarities of the base population and correspond to 0.18, 0.15, 0.14, and 0.12 constraints imposed on S and 0.40, 0.35, 0.33, and 0.3 constraints imposed on K, respectively.

Abbreviations: BV, breeding values; Index, combination of BV and Mendelian sampling variance; K, standardised similarity matrix; MOCS, Mendelian sampling‐based optimal contribution selection; S, similarity matrix.

*Selection Criteria*: Under the assumption of known additive marker effects, an average genetic map, and phased genotypes, we calculated BV $b_{i}$ for each parent using Equation (1). We also computed the index $I_{i}$ for parent i following Bijma et al. (2020) as shown in Equation (19). Here, $x_{p}$ represents the standardised truncation point for the selected proportion p, and $\sigma_{g_{i}}$ is the Mendelian standard deviation (square root of MSV) of the BVs of gametes produced by parent i.

(19) $$I_{i}=b_{i}+\sqrt{2}\cdot x_{p}\cdot \sigma_{g_{i}}$$

*Selection rules*: For the TS scheme, the top 1% (5) of males and 50% (250) of females were selected based on the selection criteria and subsequently mated randomly. Each female was allowed one mating, while each male was assigned 50 females. Each mating resulted in four offspring, maintaining a constant population size of 1000 individuals. To ensure an equal number of male and female offspring, sexes were systematically assigned (i.e., male, female, male, etc.).

For MOCS, we derived similarity matrices S for all potential parents in each generation t using the gametic approach. These matrices were scaled using their maximum element (or MSV) to maintain a consistent constraint across generations. Additionally, the matrices were standardised to obtain K. When S was not positive definite, we employed the “*nearPD*” function in *Matrix* version 1.5–4 (Douglas and Maechler 2023) to approximate positive definiteness. The ‘*nearPD*’ algorithm adapts the modified alternating projections method of Higham (2002) and incorporates procedures to ensure positive definiteness (Maechler 2022).

Subsequently, we determined the optimal contributions of parents $n_{t}$ for the next generation under varying constraints on average haplotype similarity ${\bar{Q}}_{t+1}$, as detailed in Table 2. This optimisation problem in Equation (18) was solved using the *gurobi* package version 10.0–0 (Gurobi Optimisation and LCC 2022). Constraints were established at fixed levels based on the 60%, 40%, 30%, and 20% quantiles of the haplotype similarities observed in the base population. These constraints are consistently applied across all generations to mirror the diversity levels initially established in the base population.

Since females have a limited number of offspring, the top 50% (250) of females were selected based on either BVs or indices, each allocated to a single mating. For males, mating opportunities were determined by their optimised contributions, restricting their contribution to a maximum of one male per 50 females while ensuring a minimum of five males were available for mating. This aligns with the protocols used in TS scenarios based on BVs and indices. The operational process involved creating a vector of male identifiers, each repeated according to its contributions from the optimisation. This vector was then randomised to assign males to females. The remaining conditions mirrored those in scenarios involving BVs or indices, as detailed in an earlier section. These include the number of offspring per mating, the sex ratio of the offspring, and the systematic assignment of sexes to maintain a balanced population structure.

*Parameters monitored*: Several key variables were parameters across the selection schemes and averaged over all 100 repetitions to facilitate the comparison of scheme performance. The first monitored variable is genetic gain (genetic standard deviation units), calculated as the mean increase in breeding value relative to the base population divided by the genetic standard deviation of the base population. The second monitored variable is the additive genetic variance expressed as the standard deviation of BVs for all 1000 offspring. We also monitored the level of genomic inbreeding using the formula $F_{t}=1-{\mathrm{Ho}}_{t}$, where ${\mathrm{Ho}}_{t}$ signifies the average observed heterozygosity of SNP marker loci for each generation t. Lastly, we monitored the number of favourable QTL alleles lost, the mean favourable QTL allele frequency, and the number of SNP alleles lost.

All statistical analyses were performed using programs written by the authors and packages in R version 4.2.1 (R Core Team 2022).

## 3 Results

### 3.1 Computational Improvements for MSV Prediction

Our new representation for estimating MSV ($m_{i}^{\prime }{\mathrm{Rm}}_{i}$) showcased a remarkable improvement over the previous approach by Bonk et al. (2016). We achieved a 73‐fold increase in computational speed when calculating MSVs for a set of 265 parents with 10,304 markers (refer to Data S3). This significant enhancement is vital for managing the computational demands of contemporary genomic selection programs.

### 3.2 Similarities Between Haplotypes of Parents in Holstein‐Friesian Cows

**FIGURE 1:**
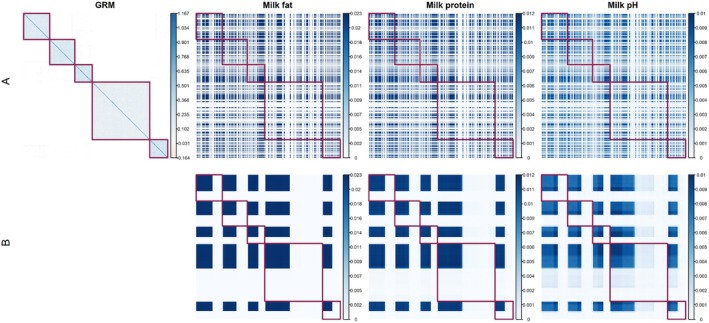
Genomic relationship matrix (GRM) and similarity matrices for all chromosomes (a) and Euclidean clustering of similarity matrices for all chromosomes (b) for milk fat, protein, and pH. The red blocks demarcate each paternal half‐sib family. Parents are arranged according to their pedigree. [Colour figure can be viewed at wileyonlinelibrary.com]

Using data from Hampel et al. (2018), we derived gametic similarity matrices and compared them with the genomic relationship matrix (GRM) as outlined by Method 1 in Raden (2008). The GRM, which is based on marker genotypes, effectively delineated family structures in the Holstein‐Friesian cow population, as shown in Figure 1. It demonstrated a heightened genetic similarity amongst progeny from the same family. In contrast, our gametic similarity matrices, based on Mendelian segregation patterns, did not exhibit such pronounced family structures in the studied traits (Figure 1A), offering a different perspective on genetic relationships. This difference arises because the GRM highlights genetic similarities within families due to shared alleles, whereas the gametic similarity matrix captures allele segregation patterns, reflecting genetic relationships that are not strictly family‐based.

The composition of our gametic similarity matrices is crucial. They consist of diagonal elements representing the MSV of each parent and off‐diagonal elements denoting similarities amongst the Mendelian sampling values of potential parents. The values in these matrices are influenced by the linkage phase, heterozygosity, genetic architecture of traits (marker effect size), and the distance between markers on chromosomes. Notably, parents with common heterozygosity of markers with large effects for a trait in the same linkage phase appeared more similar than those with small effects. Parents that were homozygous at these loci appeared less similar (numerical examples in A.3 Data S1). Consequently, individuals with comparable MSV values due to common heterozygous loci appeared similar regardless of their family.

**FIGURE 2:**
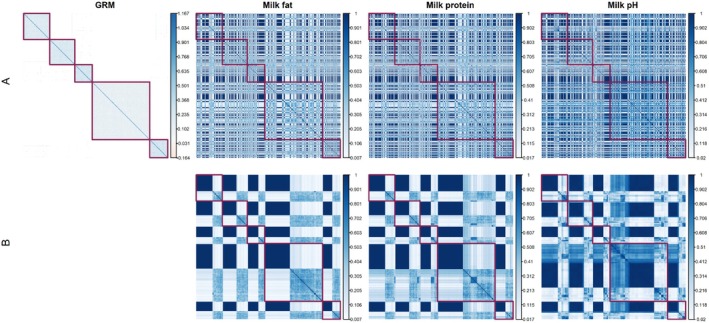
Genomic relationship matrix (GRM) and standardised similarity matrices (A) and Euclidean clustering of standardised similarity matrices (B) for milk fat, protein, and pH. The red blocks demarcate each paternal half‐sib family. Parents are arranged according to their pedigree. [Colour figure can be viewed at wileyonlinelibrary.com]

Family‐wise clustering revealed distinct similarity structures across different families (Figure 1B). We observed variations in the matrices across traits, with pairwise similarities spanning a range in milk fat (0–0.023), protein (0–0.012), and pH (0–0.01). These patterns persisted across all traits. Our standardised similarity matrices (Figure 2) further emphasised these trends but highlighted smaller similarities better, with ranges extending from 0.007 to 0.9997 in milk fat, 0.017 to 0.998 in protein, and 0.020 to 0.997 in pH.

Analysis of chromosome‐specific similarities underscored the roles of heterozygosity and genetic architecture in shaping genetic relationships. For instance, individuals in family 4 exhibited greater variability in milk fat than those in family 3, while the opposite was true for milk protein (refer to Figures S4 and S5). The contrasts in pairwise similarity values across different chromosomes highlighted the substantial impact of genetic architecture on similarity values (Figure S4). For example, in milk fat (Figure S4), pairwise similarities ranged from 0 to 0.023 on chromosome 14, while values on chromosome 4 were close to zero, suggesting that the number and effect size of markers on a chromosome significantly determine similarity values. Figure S6 presents the estimated marker effects for traits on these chromosomes and across the genome, providing insight into the effects of the distribution and effect sizes of markers on our similarity matrices.

**FIGURE 3:**
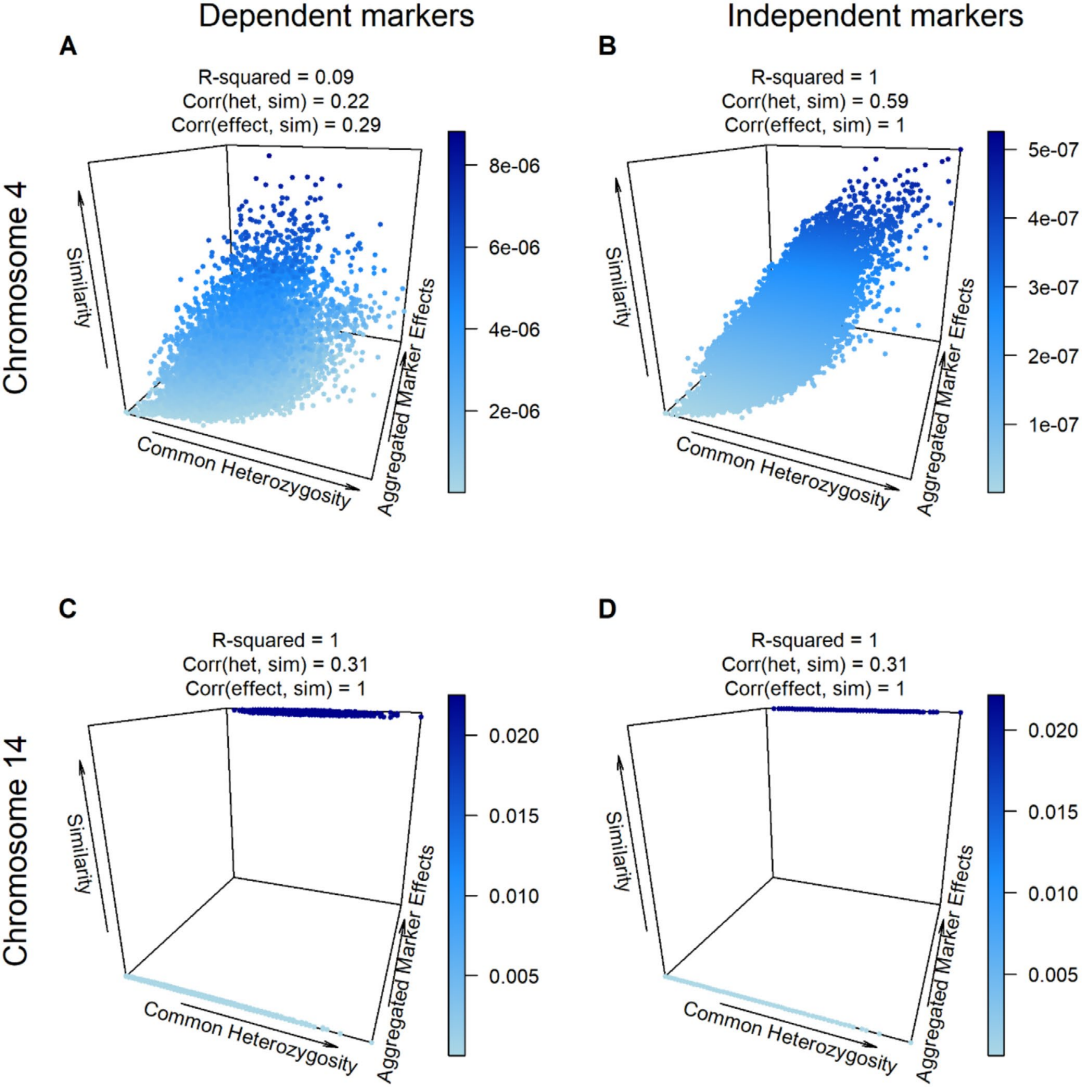
Relationship between haplotype similarities, common marker heterozygosity, and marker effect sizes in milk fat. Panels A and C depict dependent marker scenarios on chromosomes 4 and 14, respectively. Conversely, Panels B and D present independent marker scenarios on the same chromosomes, illustrating notable contrasts in relationships. [Colour figure can be viewed at wileyonlinelibrary.com]

Our linear regression analysis delved into the relationship between haplotype similarities, common marker heterozygosity, and marker effect sizes in milk fat (Figure 3). In the dependent marker scenario on chromosome 4 (Figure 3A), the analysis revealed an *R* ^2^ value of 0.09, with correlation coefficients of 0.22 for common heterozygosity and 0.29 for aggregated marker effects. A contrasting scenario emerged on chromosome 14 (Figure 3C), where the *R* ^2^ value reached 1, with correlation coefficients of 0.31 and 1 for common heterozygosity and aggregated marker effects, respectively. Under independent marker conditions, we observed enhanced prediction accuracy and correlation coefficients, emphasising the influence of heterozygosity and marker effects on haplotype similarities (Figure 3B,D). These findings were consistent across various traits and analyses, including standardised haplotype similarities (see Figures S7–S11).

### 3.3 Use of Similarity Matrices for Preserving Haplotype Diversity

**FIGURE 4:**
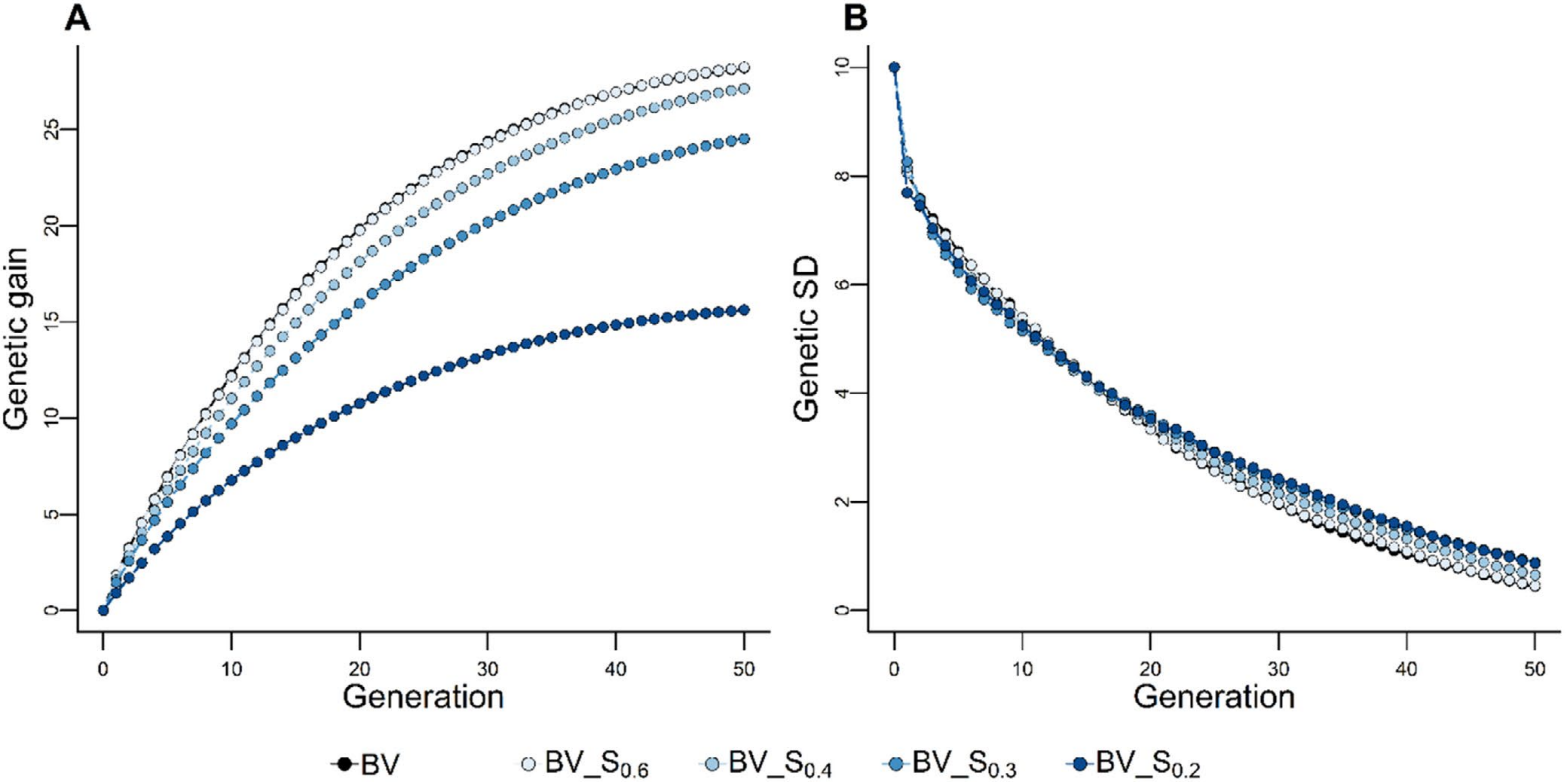
Effect of similarity matrix (S) on the cumulative genetic gain in genetic standard deviation (A) and genetic standard deviation (B). The selection schemes BV_S_0.6_ (_0.4_, _0.3_, _and 0.2_) optimise mate contribution by maximising breeding value under various constraints (0.6, 0.4, 0.3, and 0.2) on the haplotype similarity of parents. [Colour figure can be viewed at wileyonlinelibrary.com]

We evaluated the impact of using similarity matrices for MOCS on genetic gain and variance (Figure 4). In scenarios with minimal haplotype similarity constraints (e.g., BV_S_0.6_), we observed a marginally higher genetic gain of 0.04 standard deviations compared to TS on BV. Larger constraints (e.g., BV_S_0.4_, _0.3_, _and 0.2_) led to reduced genetic gains across generations, with reductions of up to 12.6 standard deviations in the last generation. However, the long‐term genetic variability preserved by MOCS schemes exceeded that of BV (Figure 4B), maintaining 0.02–0.45 standard deviations more genetic variability in the terminal generation, though at the expense of lower short‐term genetic variance.

**FIGURE 5:**
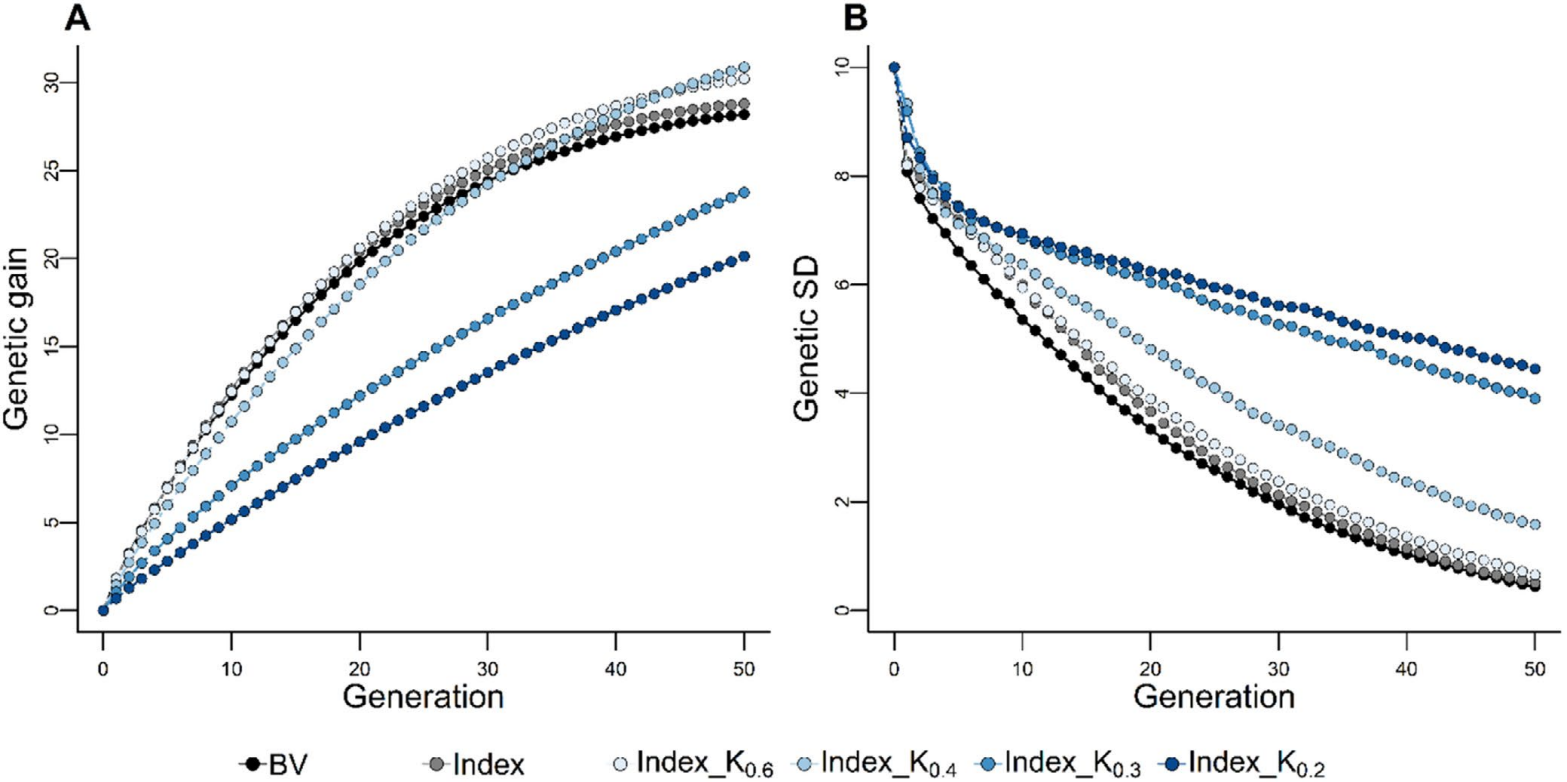
Effect of standardised similarity matrix (K) on the cumulative genetic gain in genetic standard deviation (A) and genetic standard deviation (B). The selection schemes Index_K_0.6_ (_0.4_, _0.3_, _and 0.2_) optimise mate contribution by maximising the index combining breeding value and Mendelian sampling variance under various constraints (0.6, 0.4, 0.3, and 0.2) on the standardised haplotype similarity of parents. The results maximising the breeding value are presented in Figure S12. Results are reported for 100 simulation runs. [Colour figure can be viewed at wileyonlinelibrary.com]

MOCS schemes employing standardised similarity matrices significantly impacted long‐term genetic gain, with some schemes (BV_K_0.4_, Index_K_0.4_) surpassing BV and index‐based selections by 1 and 2 standard deviations, respectively (Figures 5A and S12A). However, larger constraints in MOCS schemes sometimes resulted in up to 9 standard deviations less genetic gain. Notably, MOCS schemes with standardised matrices preserved a significantly higher range of genetic variability (0.1–4.5 standard deviations) compared to those using TS (Figure 5B and Figure S12B). The short‐term genetic variances were lower in some cases, though not as markedly as in schemes with unstandardized matrices.

**FIGURE 6:**
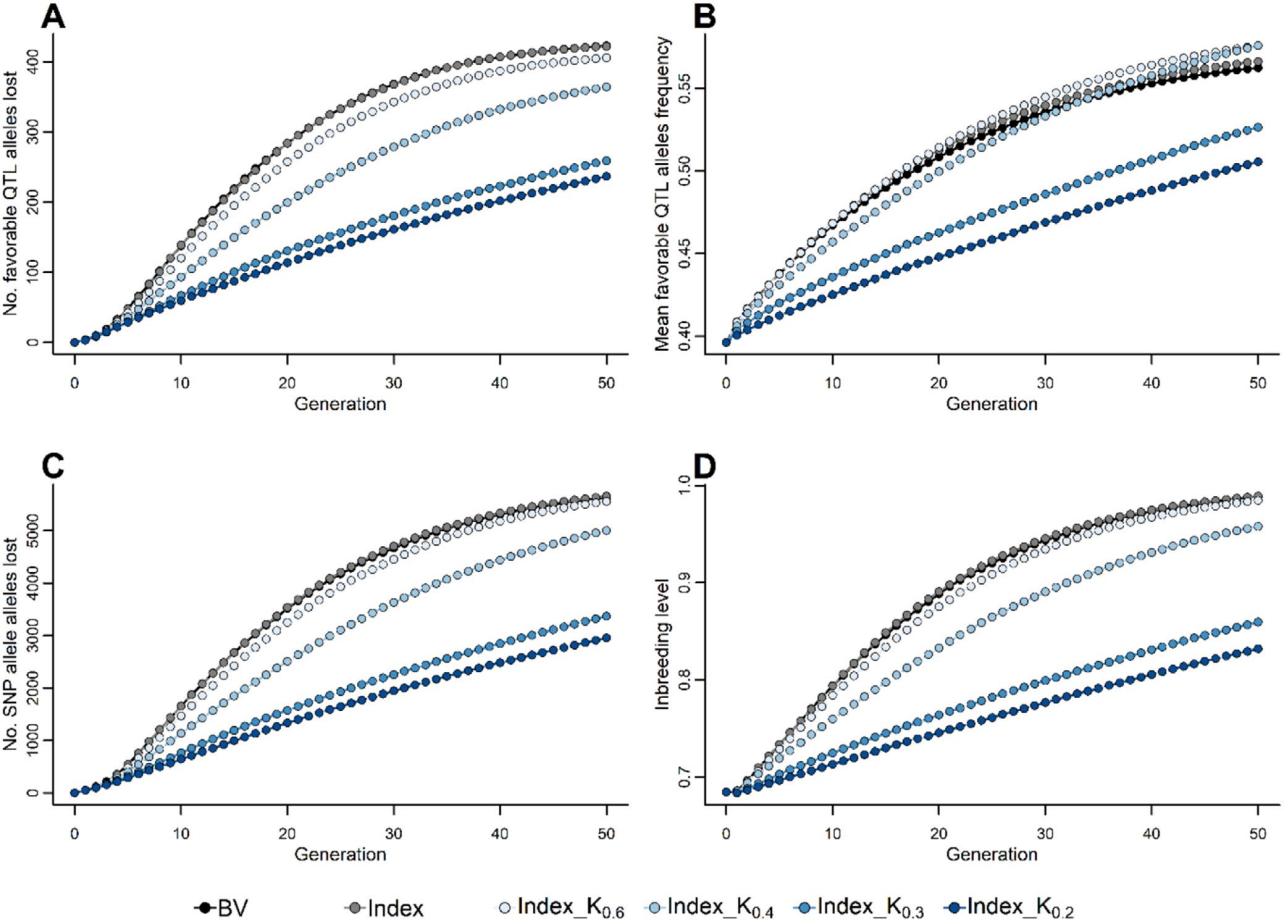
Effect of standardised similarity matrix (K) on favourable QTL alleles lost (A), mean favourable QTL allele frequency (B), SNPs lost (C), and expected inbreeding rate (D). The selection schemes Index_K_0.6_ (_0.4_, _0.3_, _and 0.2_) optimise mate contribution by maximising the index combining breeding value and Mendelian sampling variance under various constraints (0.6, 0.4, 0.3, and 0.2) on the standardised haplotype similarity of parents. The results maximising the breeding value are presented in Figure S13. Results are reported for 100 simulation runs. [Colour figure can be viewed at wileyonlinelibrary.com]

To gain a deeper understanding of the impact of similarity matrices on genetic gain and variance, we quantified favourable QTL allele loss, SNP allele loss, favourable allele frequency, inbreeding level, and the number of selected sires (Figures 6, S13,S14). MOCS schemes with standardised matrices retained (5%–44%) more favourable QTL alleles than TS schemes (Figure 6A), enhancing the frequency of these alleles beyond their TS counterparts (Figure 6B). Furthermore, these schemes preserved a greater number of SNP alleles (2%–48%) reflecting the selection and drift effect near QTL and neutral loci (Figure 6C). The inbreeding levels were also markedly different, with TS on BV leading to a higher level of inbreeding (1%) than the TS on the index, which had a 3%–16% higher level of inbreeding than MOCS schemes in the last generation (Figure 6D).

As constraints on parental haplotype similarity were increased, there was an observable increase in the number of sires chosen for mating, as detailed in Table S3. This trend led to a more diverse set of haplotypes amongst the selected parents, thereby enhancing the preservation of favourable haplotypes within the population. Notably, the MOCS schemes employing standardised similarity matrices tended to select a greater number of sires compared to those using non‐standardised matrices, reflecting a more robust approach to preserving genetic diversity.

## 4 Discussion

The similarity matrix introduced in this study addresses a key methodological gap in genomic selection, specifically focusing on the preservation of haplotype diversity amongst selected parents. This aspect is crucial when selections are informed by BVs or an index combining expected BVs with MSVs. Our matrix provides a nuanced approach to assessing genetic relationships, essential for effective breeding strategies. The practical application of our approach is highlighted by the observation of standardised similarities in real data across a spectrum of values for various traits, indicating its broad adaptability and utility. This adaptability is further evidenced by trait‐specific heterogeneity. Additionally, simulation results validate that using the matrix for parent selection not only enhances genetic diversity and phenotypic outcomes but also drives substantial genetic gains while preserving trait variability across breeding programs.

The derived similarity measure is intrinsically interpretable: high similarity between parents indicates numerous shared chromosome segments with markers exhibiting large additive effects in the same linkage phase. This phenomenon was evident in our analysis when parents shared identical haplotypes, yielding a perfect correlation in the similarity measure. While established methods like the Kullback–Leibler and Jensen–Shannon divergence are possible alternatives for comparing Mendelian sampling values (Kullback and Leibler 1951; Nielsen 2019), their applicability in breeding contexts requires further investigation.

One of the perks of our approach is the expedited prediction of MSVs. Utilising marker effects with parent‐specific signs and an expected within‐family LD matrix—a departure from approaches used by Bonk et al. (2016) and others—enhances computational efficiency, critical in managing large datasets. This efficiency is maintained even when adapting our methodology to scenarios involving many traits and fewer parents, as the process of adjusting marker effects to parent‐specific vectors is straightforward and efficient. The effectiveness of our similarity matrices relies on the availability and accuracy of phased genotypes, genetic maps, and marker effect estimates, and they can be adapted for multi‐trait selections using index weights.

Using the Holstein‐Friesian dataset, we demonstrated the capability of these similarity matrices to provide a comprehensive view of Mendelian sampling values, both within and across families. Our results showed significant variation in genetic relationships, influenced by factors like linkage, heterozygosity in parents, and trait genetic architecture, as detailed in Figures 1, 2 and S4, S5. Standardised similarities, representing the proportion of shared MSV, spanned a wide parameter space (Figure 2), suggesting a need for further research into potential variations amongst different species and populations.

Our linear regression analysis of Holstein‐Friesian cows unveiled the substantial impact of both heterozygosity and marker effects on haplotype similarities, as depicted in Figure 3. The notably improved prediction accuracy in scenarios with independent markers, relative to those with dependent markers, can be primarily attributed to the model's emphasis on heterozygosity. Conversely, the model's diminished predictive performance in the dependent marker scenario brings to the fore the complexities of linkage phases. These intricacies are pivotal for understanding genetic inheritance patterns, which were not considered by the prediction model. Nevertheless, our analysis consistently underlines the profound influence of heterozygosity and marker effects. Specifically, it highlights how similarities between parents increase with an increase in the number of shared chromosomal segments that have markers exerting significant additive effects on the trait. This correlation corroborates the observations from Figures 1, 2, and the detailed examples in A.3 Data S1 further illuminate the relationship between common heterozygosity and specific allele contributions to haplotype similarities. These insights are instrumental in selecting parent pairs that not only maximise genetic gains but also preserve essential genetic diversity.

Our approach utilises a similarity matrix that incorporates within‐family LD and marker effects. By doing so, it refines the genomic selection processes and eliminates the need for segmental GEBVs (Kemper et al. 2012; Daetwyler et al. 2015). This approach is particularly advantageous in high selection intensity scenarios, adeptly balancing the preservation of haplotype diversity with genetic gain enhancement. Moreover, in breeding programs with lower selection pressure, our approach demonstrates comparable efficacy to traditional TS, highlighting its adaptability and effectiveness in various breeding contexts.

To evaluate the effectiveness of similarity matrices in preserving haplotype diversity, we analysed various selection schemes. This included combinations of BVs or indices with TS or MOCS rules. As anticipated, TS applied to the index outperformed BV in terms of longer‐term genetic gain and variance preservation. This can be attributed to the index's ability to achieve higher average frequencies of favourable QTL alleles, as evidenced in Figure 6B, and consistent with previous simulation studies (Müller et al. 2018; Allier et al. 2019; Musa and Reinsch 2022). While the index method achieves higher favourable QTL allele frequencies, the near‐identical numbers of QTL and SNP alleles lost across both selection methods, along with comparable inbreeding levels (as shown in Figure 6), can be explained by the biallelic nature of the markers and the random mating strategy employed. This setup means that the number of SNPs lost is equivalent to the observed genomic inbreeding or homozygosity, which in turn equalises genetic drift across the selection methods.

Furthermore, MOCS schemes surpassed their TS counterparts in long‐term genetic gain and variance, though this was at the cost of reduced short‐term genetic gain (Figures 5 and S12). This result aligns with the expected trend in long‐term genomic selection strategies, which prioritise genetic diversity and long‐term gains over immediate outcomes (Daetwyler et al. 2015; Liu et al. 2015; Müller et al. 2018; Allier et al. 2019). Notably, MOCS schemes exhibited lower short‐term genetic variance (Figure 4B), a finding attributable to the nuances of the similarity measure used.

The similarity measure is intended to prevent the selection of parents with high MSV potential caused by similar chromosomal segments, thus avoiding the unintentional loss of favourable haplotypes when selection is based on the index or BVs. As a result, constraining the haplotype similarity of parents may therefore sacrifice the selection of parents with large MSVs caused by favourable haplotypes in coupling with large effects on the trait(s) of interest in favour of parents with lower MSVs resulting from different chromosomal segments. The former could recombine in later generations to produce high genetic variability. In contrast, despite having parents with more diverse haplotypes, the latter could produce lower genetic variability in initial generations, as evidenced in Figure 4B.

On the other hand, constraining the standardised similarity measure emphasises the similarity between parental haplotypes rather than MSVs, allowing a broader range of favourable haplotypes to remain viable over successive generations (Figures 6A and S12A). Consequently, up to 2 and 4.5 standard deviations more long‐term genetic gain and variability were achieved than TS counterparts (Figures 5 and S12), as opposed to constraining the unstandardized similarity measure, which led to only up to 0.04 and 0.45 standard deviations more genetic gain and genetic variability in the last generation. The standardised similarity matrix also results in a less conspicuous lower short‐term genetic variance (Figures 5B and S12B) compared to the unstandardized version.

The OCS (Meuwissen and Sonesson 1998; Sonesson et al. 2012; Woolliams et al. 2015) and MOCS strategies aim to optimise parental contributions to the next generation by maximising genetic gain under a given constraint on the average similarity between parents. Their primary distinction lies in the matrices used to impose these constraints. OCS employs a GRM based on parental genotypes, which assesses genetic relationships independently of any specific traits. In contrast, MOCS uses a similarity matrix based on Mendelian segregation patterns, derived using trait‐specific marker effects estimates. Therefore, these similarity measures address different aspects of diversity and serve distinct purposes, making them suitable for different breeding goals and scenarios.

OCS has been shown to either maintain allele frequencies close to the original population or shift them towards 0.5, depending on the GRM used (Gómez‐Romano et al. 2016; Meuwissen et al. 2020). Given that genetic improvement is driven by increasing the frequency of favourable alleles, OCS may not effectively maximise short‐ and mid‐term genetic gain and could lead to the accumulation of deleterious alleles in the genome, thus reducing population fitness (de Cara et al. 2013). Consequently, a similarity matrix indicating the extent to which parents share heterozygous QTL segments, as used in MOCS schemes, may be a more suitable similarity measure when the goal is to maximise genetic gain while preserving genetic diversity.

To validate this hypothesis, we compared our results with those of optimisations involving GRM, henceforth referred to as OCS schemes. We developed additional selection schemes with $Q_{t}$ as GRM and constraints of 1% and 0.5% on the average rate of inbreeding (see Equation 18). GRMs were derived according to Method 1 of Raden (2008), and a constraint was imposed to select a minimum of five males for mating. Like MOCS schemes, only males were optimised in OCS schemes, while females were selected based on either TS on BV or index. We observed that OCS generally led to greater long‐term genetic gain and preserved more genetic variability than MOCS schemes, albeit at the expense of short‐term genetic gain (Figure S15). However, the MOCS scenario with the most stringent constraint on standardised similarities preserved more genetic diversity in the long term compared to OCS schemes. This finding suggests that the various facets of diversity accounted for by the matrices significantly influence the selection outcomes. While OCS, with its focus on controlling inbreeding, led to the selection of a larger number of sires (mean of 40–72 sires) to achieve the desired rate of inbreeding compared to MOCS schemes (mean of 7–29 sires across schemes), which selected fewer sires to achieve the desired haplotype similarity (Table S4).

We further set the additional constraints such that the minimum and maximum number of selected sires were 5 and 25. Here for both OCS and MOCS, we adapted these constraints iteratively, particularly in instances where the number of selected sires surpassed our maximum threshold. This adjustment process continued until the number of selected parents fell within our specified range. Our findings, as detailed in Table S4, indicated that OCS and the most restrictive MOCS scheme chose a similar number of parents. However, a critical difference emerged in their outcomes: the MOCS scheme was more effective in preserving genetic variability than OCS (Figure S16). This result underscores the strength of the MOCS approach, particularly its ability to identify key parents for breeding programs, while also emphasising the importance of maintaining genetic diversity.

While the benefits of our approach are clear, particularly in selecting fewer parents than OCS while preserving comparable haplotype diversity, there is significant potential for further improvement. Future efforts could focus on refining this approach by adjusting constraints, promoting non‐random mating, and optimising mate allocation for both males and females. Comparative effectiveness studies are suggested to evaluate the GRM and trait‐specific similarity matrices under diverse breeding conditions, considering physiological, logistic, and non‐additive genetic effects.

Given that our research often relies on known values for BVs and marker effects—unlike typical real‐world settings—it is critical to validate this approach under realistic conditions. This validation should involve assessing the performance of the similarity matrix when genetic parameters are estimated and conducting comprehensive testing in practical breeding environments. Moreover, as breeding objectives evolve due to environmental changes, market demands, and regulatory shifts, the neutrality and flexibility of the GRM may become increasingly important. Offering a neutral measure of genetic relationships, the GRM may robustly manage genetic risks in scenarios with poorly estimated genetic values. Future studies should not only explore various genetic architectures and multi‐trait selections to assess the matrix's effectiveness—especially its impact on the accumulation of deleterious alleles and overall genetic health and viability—but also compare the performance of similarity matrices and GRM in these dynamic environments.

Recommending constraints for OCS is straightforward (0.5%–1% inbreeding rates for livestock populations as per FAO 2015; Meuwissen and Oldenbroek 2017), but doing so for MOCS is more complex. This complexity arises from the dependence of the similarity matrices' values on factors like genetic architecture and the number of traits under selection, unlike GRM, which is based on genome‐wide similarities.

Another technical aspect worth mentioning involves addressing an indefinite similarity matrix when encountered. This may arise due to rank deficiencies in the family‐specific marker effects vector $m_{i}$. Recall that homozygous loci in $m_{i}$ vectors are set to zero, and heterozygous markers retain their effects but vary in sign based on the linkage phase. Consequently, heterozygous loci with zero marker effects also result in zero. This situation, where the number of parents exceeds the number of informative markers, is amplified in chromosome‐specific calculations, posing a significant challenge for optimisation algorithms that require a positive definite matrix. This challenge is detailed in Table S5. Additional contributing factors include parent heterozygosity, genetic trait architecture, marker effects estimation method, population type, and the number of traits under selection.

This study focused on applying the similarity matrices in optimising contributions to the next generations when genotypic data from both male and female parents are available. However, they can also be used to optimise mating decisions in scenarios where genotypic data are only available for one sex. Another potential application of gametic similarity matrices is plant breeding, where gametes from parents are converted into double‐haploid lines that form the next breeding generation.

In conclusion, we derived trait‐specific similarities between parents reflecting the shared heterozygosity of their markers with large effects and similar linkage phases. The similarity of a parent to itself is equal to its MSV. When combined in a matrix, these similarities provide the opportunity to balance haplotype diversity and genetic gain in selection decisions. Combining the similarity matrix and breeding value or index preserved genetic variability in a simulated breeding program more effectively than selection based on breeding value and index alone without compromising genetic gain.

## Conflicts of Interest

The authors declare no conflicts of interest.

## Supporting information


Data S1.

Data S2.

Data S3.

Data S4.

Data S5.

## Data Availability

The datasets supporting the conclusions of this article are included in the article and its Supporting Information. The pedigree information, genotypic data, and physical map of the empirical dataset can be found here: DOI: https://doi.org/10.3389/fgene.2018.00186 under the Supporting Information section. All empirical and simulated data, results, and corresponding R scripts are available at https://doi.org/10.22000/1055.

## Appendix A

### A.1 Illustration of Equivalence

Equations (2) and (5) are equivalent. To demonstrate their equivalence, consider the following case of two biallelic marker loci on a single chromosome with haplotypes in coupling and repulsion phases.

According to Bonk et al. (2016), for any two‐marker interval, we have the following covariance matrices:

$R_{a}=\left[\begin{array}{cc} 0.25 & \rho \\ \rho & 0.25 \end{array}\right]$ and $R_{b}=\left[\begin{array}{cc} 0.25 & -\rho \\ -\rho & 0.25 \end{array}\right]$.

Meanwhile, the MSVs are as follows:

(A1)
$$m^{\prime }R_{a}m=\left[\begin{array}{cc} m_{1} & m_{2} \end{array}\right]\cdot \left[\begin{array}{cc} 0.25 & \rho \\ \rho & 0.25 \end{array}\right]\cdot \left[\begin{array}{c} m_{1}\\ m_{2} \end{array}\right]=0.25\cdot m_{1}^{2}+0.25\cdot m_{2}^{2}+2\rho m_{1}m_{2}$$

(A2)
$$m^{\prime }R_{b}m=\left[\begin{array}{cc} m_{1} & m_{2} \end{array}\right]\cdot \left[\begin{array}{cc} 0.25 & -\rho \\ -\rho & 0.25 \end{array}\right]\cdot \left[\begin{array}{c} m_{1}\\ m_{2} \end{array}\right]=0.25\cdot m_{1}^{2}+0.25\cdot m_{2}^{2}-2\rho m_{1}m_{2}$$

After setting up $m_{a}$ and $m_{b}$, the same results can be obtained by setting up a population covariance matrix

$$R=\left[\begin{array}{cc} 0.25 & \rho \\ \rho & 0.25 \end{array}\right]$$

and applying Equation (5):

(A1)
$$m_{a}^{\prime }{\mathrm{Rm}}_{a}=\left[\begin{array}{cc} m_{1} & m_{2} \end{array}\right]\cdot \left[\begin{array}{cc} 0.25 & \rho \\ \rho & 0.25 \end{array}\right]\cdot \left[\begin{array}{c} m_{1}\\ m_{2} \end{array}\right]=0.25\cdot m_{1}^{2}+0.25\cdot m_{2}^{2}+2\rho m_{1}m_{2}$$

(A2)
$$m_{b}^{\prime }{\mathrm{Rm}}_{b}=\left[\begin{array}{cc} m_{1} & -m_{2} \end{array}\right]\cdot \left[\begin{array}{cc} 0.25 & \rho \\ \rho & 0.25 \end{array}\right]\cdot \left[\begin{array}{c} m_{1}\\ -m_{2} \end{array}\right]=0.25\cdot m_{1}^{2}+0.25\cdot m_{2}^{2}-2\rho m_{1}m_{2}$$

The equivalence of the two representations of MSV generalises to any number of markers (see Appendix B for proof of equivalence).

## Appendix B

### Proof of Equivalence

In Equation (2) $m^{\prime }R_{i}m=\sum_{k}m_{k}^{2}+\sum_{k}\sum_{l}m_{k}m_{l}\rho_{\mathrm{kl}}$ each $\rho_{\mathrm{kl}}$ is positive if the marker alleles to which the effects $m_{k}$ and $m_{l}$ have been assigned are on the same chromosome (in coupling). Otherwise, $\rho_{\mathrm{kl}}$ is negative. As the marker effects do not depend on the linkage phase, each product $m_{k}m_{l}\rho_{\mathrm{k1}}$ changes its sign according to the linkage phase of the respective markers.

In Eq Error! Reference source not found $m_{i}^{\prime }{\mathrm{Rm}}_{i}=\sum_{k}{\left(m_{k}^{i}\right)}^{2}+\sum_{k}\sum_{l}m_{k}^{i}m_{l}^{i}\rho_{\mathrm{kl}}$ the covariance $\rho_{\mathrm{kl}}$ is always positive, but the signs of the marker effects depend on the linkage phase. In coupling, they are as displayed in Equation (2), yet in the linkage phase, they are either $\left(m_{k},-m_{l}\right)$ or $\left(-m_{k},m_{l}\right)$ depending on the alleles on the first haplotype. In effect, this causes the sign of each product $m_{k}^{i}m_{l}^{i}\rho_{\mathrm{kl}}$ to change according to the linkage phase as before and $m^{\prime }R_{i}m=m_{i}^{\prime }{\mathrm{Rm}}_{i}$. Thus, it is quicker to set up $m_{i}$ with M elements on a chromosome than $\frac{1}{2}M\left(M-1\right)$ off‐diagonal elements of a half‐stored R‐matrix.

## Appendix C

### Monoecious Species and Order of Parents in Dioecious Organisms

In monoecious plants, parents i and j may swap their function as male (pollen donor) and female parents when producing offspring. The MSVs of the resulting zygotes then remain the same, assuming equal recombination rates in male and female parents. With sex‐specific covariance matrices $R_{m}$ and $R_{f}$ reflecting maps of males and females, the MSVs for parents ij and ji are

$$\mathrm{var}\left(g_{\mathrm{ij}}\right)=m_{i}^{\prime }R_{m}m_{i}+m_{j}^{\prime }R_{f}m_{j}\;\text{and}\;\mathrm{var}\left(g_{\mathrm{ji}}\right)=m_{i}^{\prime }R_{f}m_{i}+m_{j}^{\prime }R_{m}m_{j}$$

For two pairs of parents ij and ji there are, in principle, four different conditional covariances, depending on which individual acts as pollen donor within each pair:

$$\begin{array}{l} \text{ccov}\left(g_{\mathrm{ij}},g_{\mathrm{uv}}\right)=m_{i}^{\prime }R_{m}m_{u}+m_{j}^{\prime }R_{f}m_{v},\;\text{ccov}\left(g_{\mathrm{ji}},g_{\mathrm{vu}}\right)=m_{j}^{\prime }R_{m}m_{v}+m_{i}^{\prime }R_{f}m_{u},\\ \text{ccov}\left(g_{\mathrm{ij}},g_{\mathrm{vu}}\right)=m_{i}^{\prime }R_{m}m_{v}+m_{j}^{\prime }R_{f}m_{u}\;\text{and}\;\text{ccov}\left(g_{\mathrm{ji}},g_{\mathrm{uv}}\right)=m_{j}^{\prime }R_{m}m_{u}+m_{i}^{\prime }R_{f}m_{v}. \end{array}$$

If all four kinds of parentage have equal probabilities and $R=\frac{1}{2}\left(R_{m}+R_{f}\right)$, then the average of these four conditional covariances can be simplified as

$$\frac{1}{2}\left(m_{i}^{\prime }{\mathrm{Rm}}_{u}+m_{j}^{\prime }{\mathrm{Rm}}_{v}\right)+\frac{1}{2}\left(m_{i}^{\prime }{\mathrm{Rm}}_{v}+m_{j}^{\prime }{\mathrm{Rm}}_{u}\right)$$

Then, by taking absolute values in a chromosome‐wise manner as described above, this average conditional covariance defines the similarity ${\bar{s}}_{\mathrm{ij},\mathrm{uv}}$ between parents ij and uv as

$${\bar{s}}_{\mathrm{ij},\mathrm{uv}}=\frac{1}{2}\left(s_{\mathrm{ij},\mathrm{uv}}+s_{\mathrm{ij},\mathrm{vu}}\right)$$

This could also be used as an alternative similarity measure in mammals and dioecious plants if the similarity is to be independent of the order of parents—or, equivalently, independent of the parental origin of the haplotypes in the offspring.
