# Supplementary material for: A Similarity Matrix for Preserving Haplotype Diversity Amongst Parents in Genomic Selection
Source: J Anim Breed Genet. 2025 Mar 4;142(6):652–68. doi: 10.1111/jbg.12930 (PMC12501756; doi:10.1111/jbg.12930)
Supplement: Supplementary file 3 — Data S3. [file JBG-142-652-s003.docx]

**TABLES**

| **Table S3: Summary Statistics for the number of selected males by various selection schemes** | | | |
| --- | --- | --- | --- |
| **Scheme*** | **Minimum** | **Mean** | **Maximum** |
| **MOCS schemes involving similarity matrix** | | | |
| BV_S_0.6_ | 5 | 5.6 | 10 |
| BV_S_0.4_ | 5 | 7.8 | 17 |
| BV_S_0.3_ | 5 | 9.5 | 18 |
| BV_S_0.2_ | 6 | 12.1 | 24 |
|  |  |  |  |
| **MOCS schemes involving standardized similarity matrix** | | | |
| BV_K_0.6_ | 5 | 6.7 | 16 |
| BV_K_0.4_ | 7 | 15.4 | 35 |
| BV_K_0.3_ | 11 | 26.2 | 41 |
| BV_K_0.2_ | 16 | 28.2 | 41 |
| Index_K_0.6_ | 5 | 7.7 | 19 |
| Index_K_0.4_ | 7 | 17 | 31 |
| Index_K_0.3_ | 13 | 27.2 | 41 |
| Index_K_0.2_ | 16 | 28.6 | 42 |
| *Truncation selection on BV and index always selected 5 males. MOCS = Mendelian sampling-based optimal contribution selection; BV = breeding values; Index = combination of BV and Mendelian sampling variance; S = similarity matrix; K = standardized similarity matrix. The indices 0.6, 0.4, 0.3 and 0.2 represent the 60th, 40th, 30th and 20th percentiles of haplotype similarities of the base population and correspond to 0.18, 0.15, 0.14 and 0.12 constraints imposed on S and 0.40, 0.35, 0.33 and 0.3 constraints imposed on K, respectively. | | | |

| **Table S4: Summary Statistics for the number of selected males by various selection schemes** | | | | | | | | |
| --- | --- | --- | --- | --- | --- | --- | --- | --- |
| **Scheme*** | **Minimum** | |  | **Mean** | |  | **Maximum** | |
| **Analysis** | **5** | **5-25** |  | **5** | **5-25** |  | **5** | **5-25** |
| **Mendelian sampling-based optimal contribution selection** | | | | | | | | |
| BV_K_0.6_ | 5 | 5 |  | 6.7 | 6.6 |  | 16 | 16 |
| Index_K_0.6_ | 5 | 5 |  | 7.7 | 7.7 |  | 19 | 16 |
| BV_K_0.2_ | 16 | 15 |  | 28.2 | 23.0 |  | 41 | 25 |
| Index_K_0.2_ | 16 | 15 |  | 28.6 | 23.2 |  | 42 | 25 |
|  |  |  |  |  |  |  |  |  |
| **Optimum contribution selection** | | | | | | | | |
| BV_G_0.01_ | 26 | 19 |  | 39.6 | 24.1 |  | 58 | 25 |
| Index_G_0.01_ | 26 | 20 |  | 39.5 | 24.1 |  | 58 | 25 |
| BV_G_0.005_ | 54 | 20 |  | 70.6 | 24.1 |  | 92 | 25 |
| Index_G_0.005_ | 51 | 19 |  | 71.8 | 24.1 |  | 92 | 25 |
| *Truncation selection on BV and index always selected 5 males. BV = breeding values; Index = combination of BV and Mendelian sampling variance; K = standardized similarity matrix; G = genomic relationship matrix. The indices 0.6 and 0.2 represent the 60^th^ and 20th percentiles of standardized haplotype similarities of the base population and correspond to 0.40 and 0.30 constraints imposed, respectively. The indices 0.01 and 0.005 are the constraints imposed on G. The analysis labeled '5' corresponds to scenarios where a minimum of 5 sires were chosen as parents, without imposing an upper limit on their number. On the other hand, the '5-25' analysis pertains to scenarios with specific constraints, setting both the minimum and maximum numbers of sires to be selected at 5 and 25, respectively. | | | | | | | | |

**FIGURES**


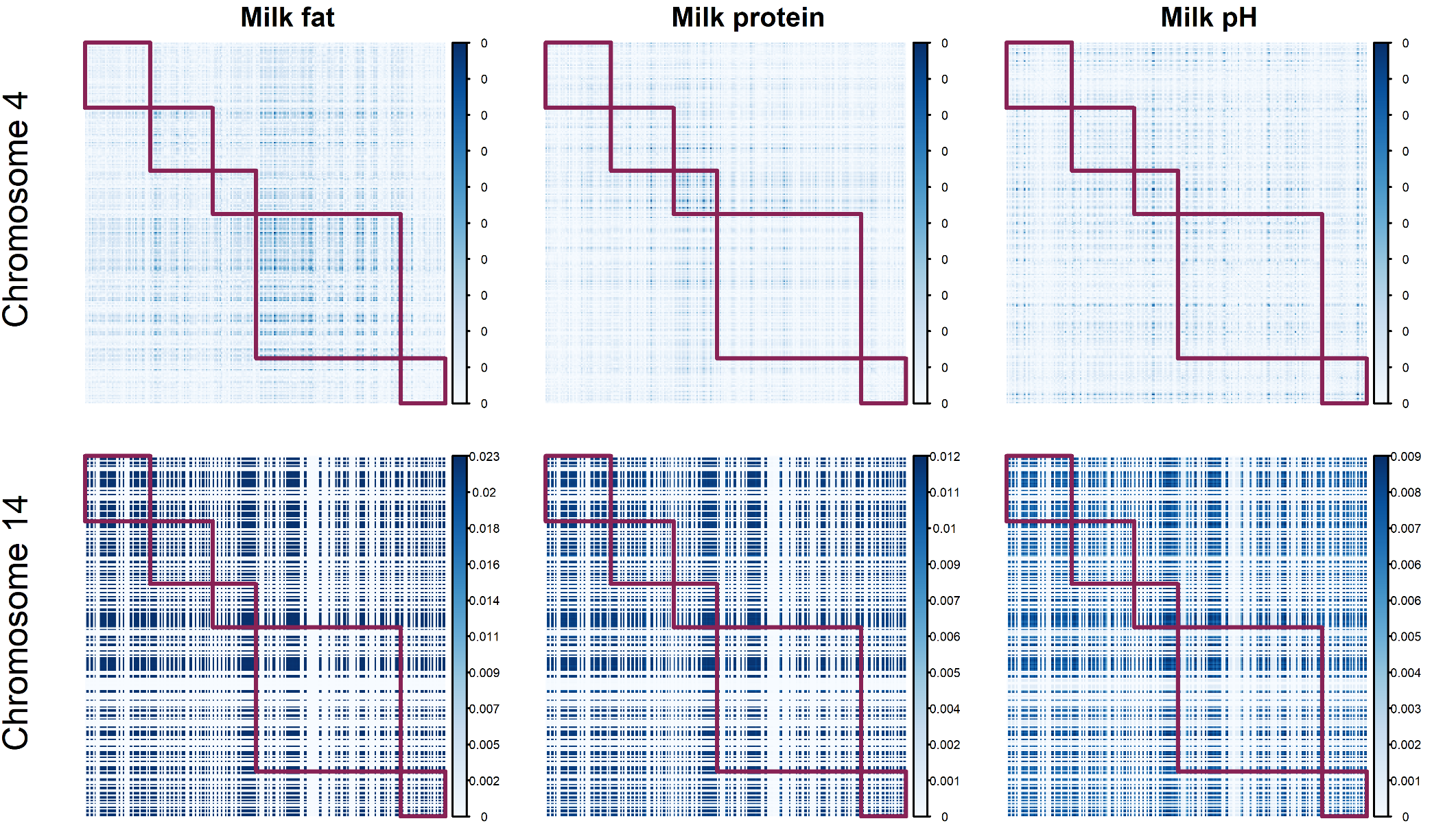


**Figure S4** Similarity matrices showing chromosomes 4 and 14 for milk fat, protein, and pH. The red blocks demarcate each paternal half-sib family. Parents are arranged according to their pedigree.


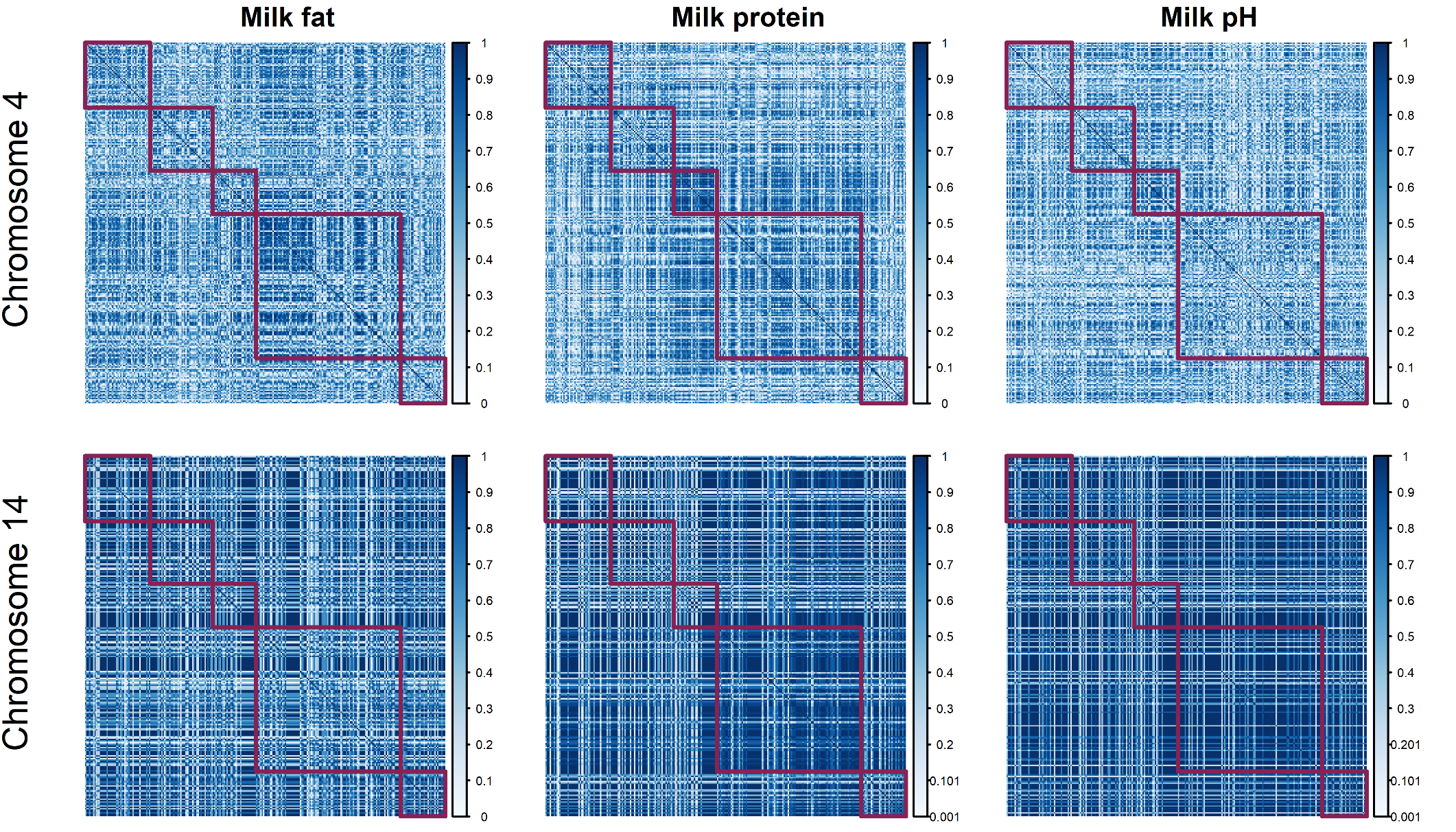


**Figure S5** Standardized similarity matrices showing chromosomes 4 and 14 for milk fat, protein, and pH. The red blocks demarcate each paternal half-sib family. Parents are arranged according to their pedigree.


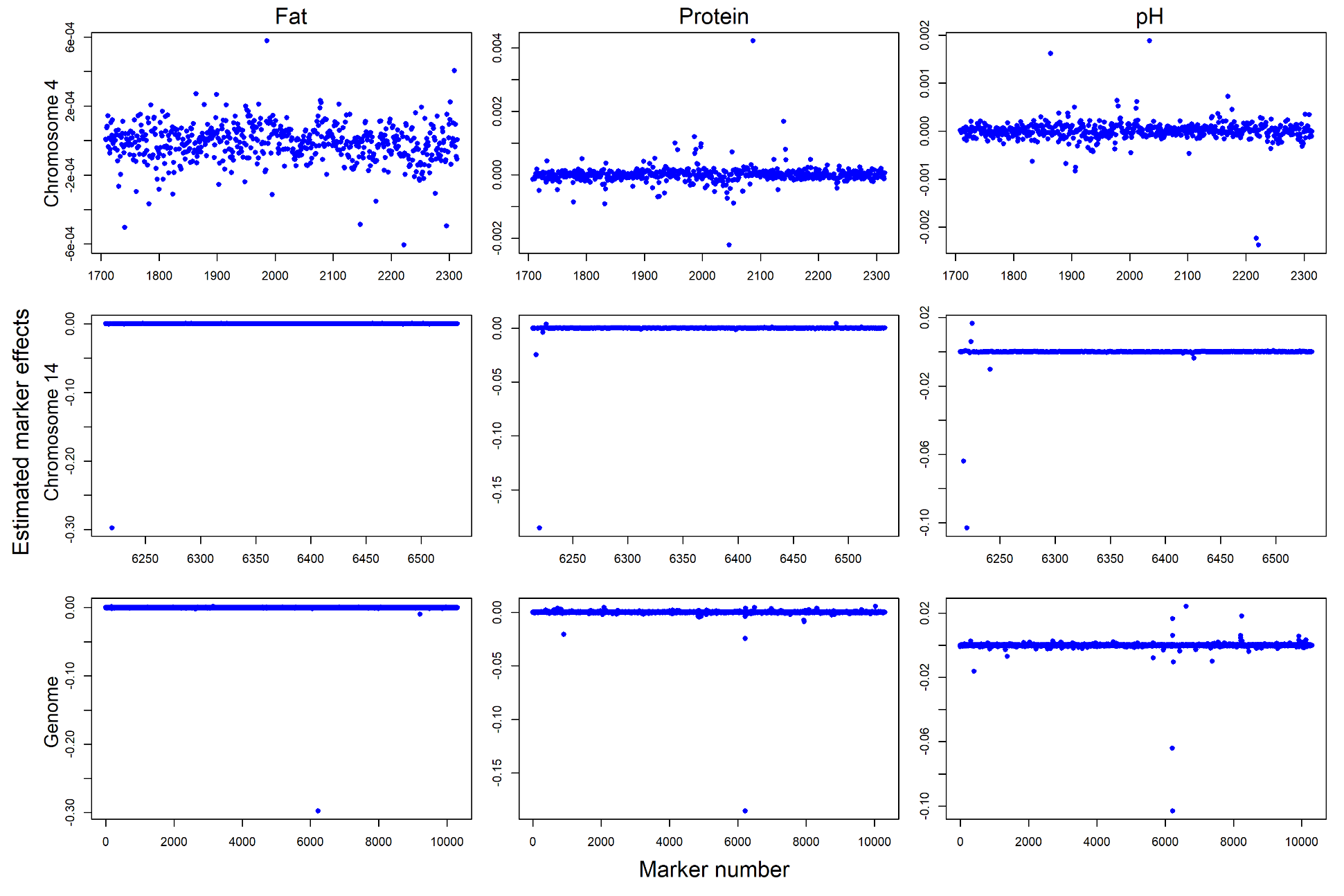


**Figure S6** Estimated marker effects across chromosomes 4 and 14, and the genome for milk fat, protein, and pH.


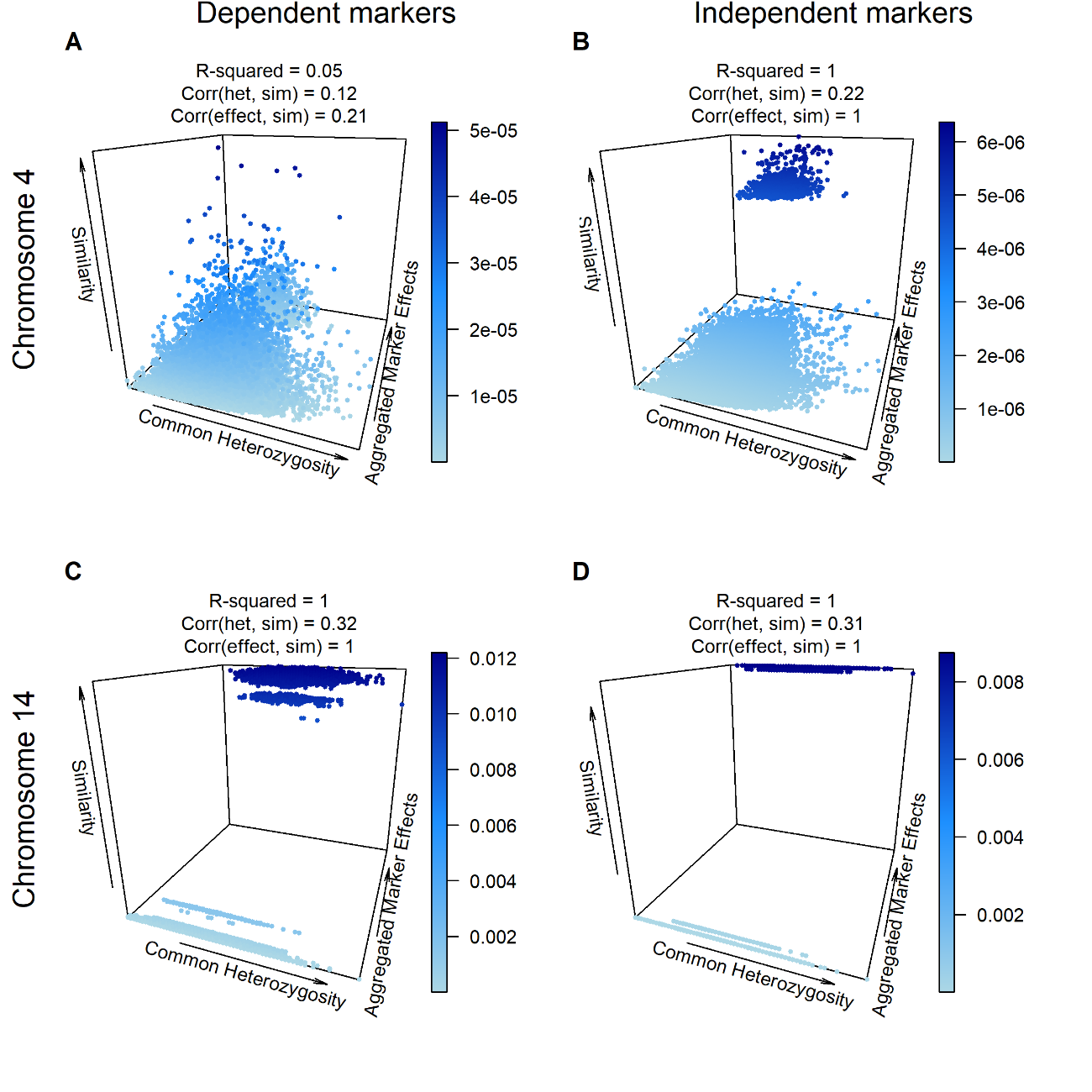


**Figure S7** Relationship between haplotype similarities, common marker heterozygosity, and marker effect sizes in milk protein. Panels A and C depict dependent marker scenarios on chromosomes 4 and 14, respectively. Conversely, Panels B and D present independent marker scenarios on the same chromosomes, illustrating notable contrasts in relationships.


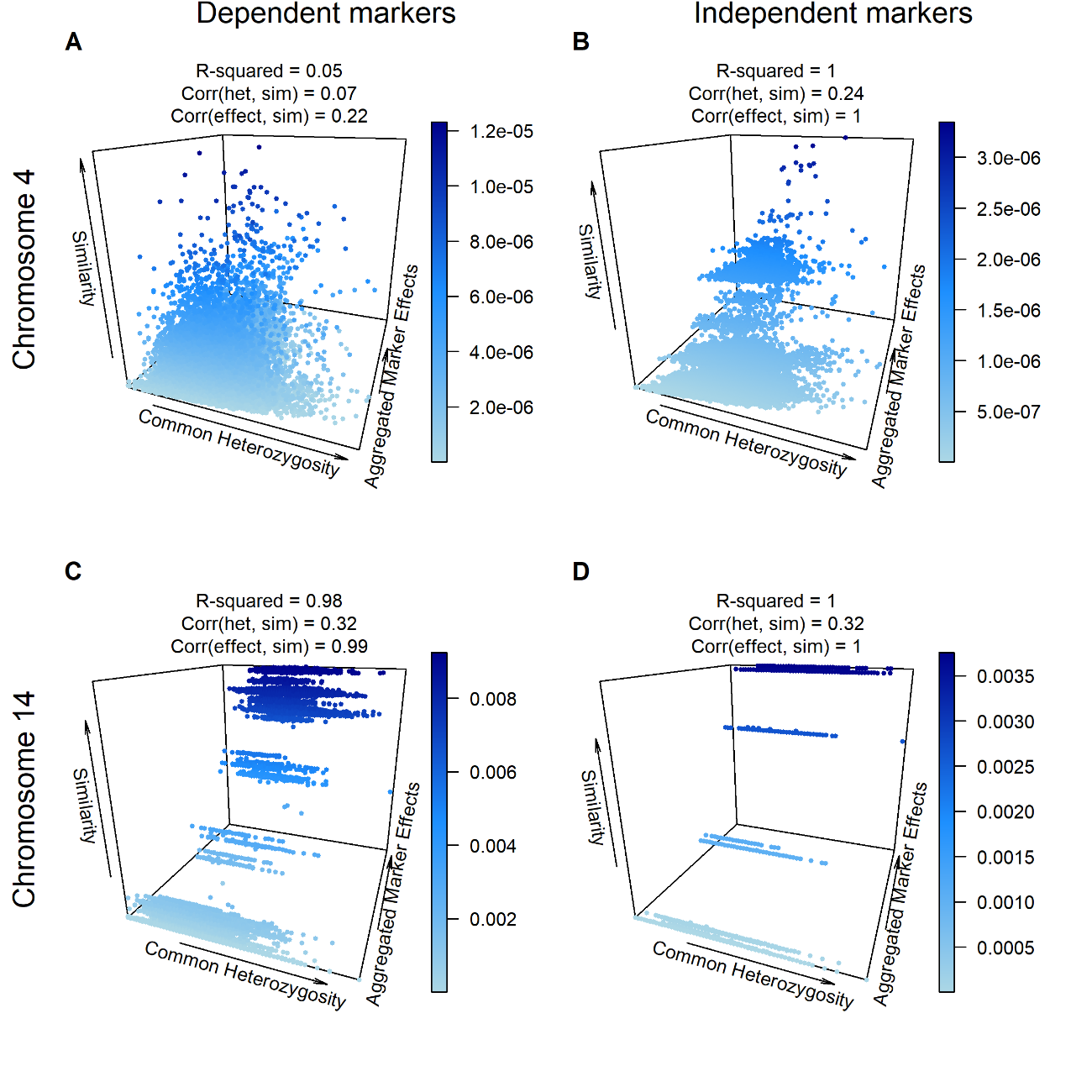


**Figure S8** Relationship between haplotype similarities, common marker heterozygosity, and marker effect sizes in milk pH. Panels A and C depict dependent marker scenarios on chromosomes 4 and 14, respectively. Conversely, Panels B and D present independent marker scenarios on the same chromosomes, illustrating notable contrasts in relationships.


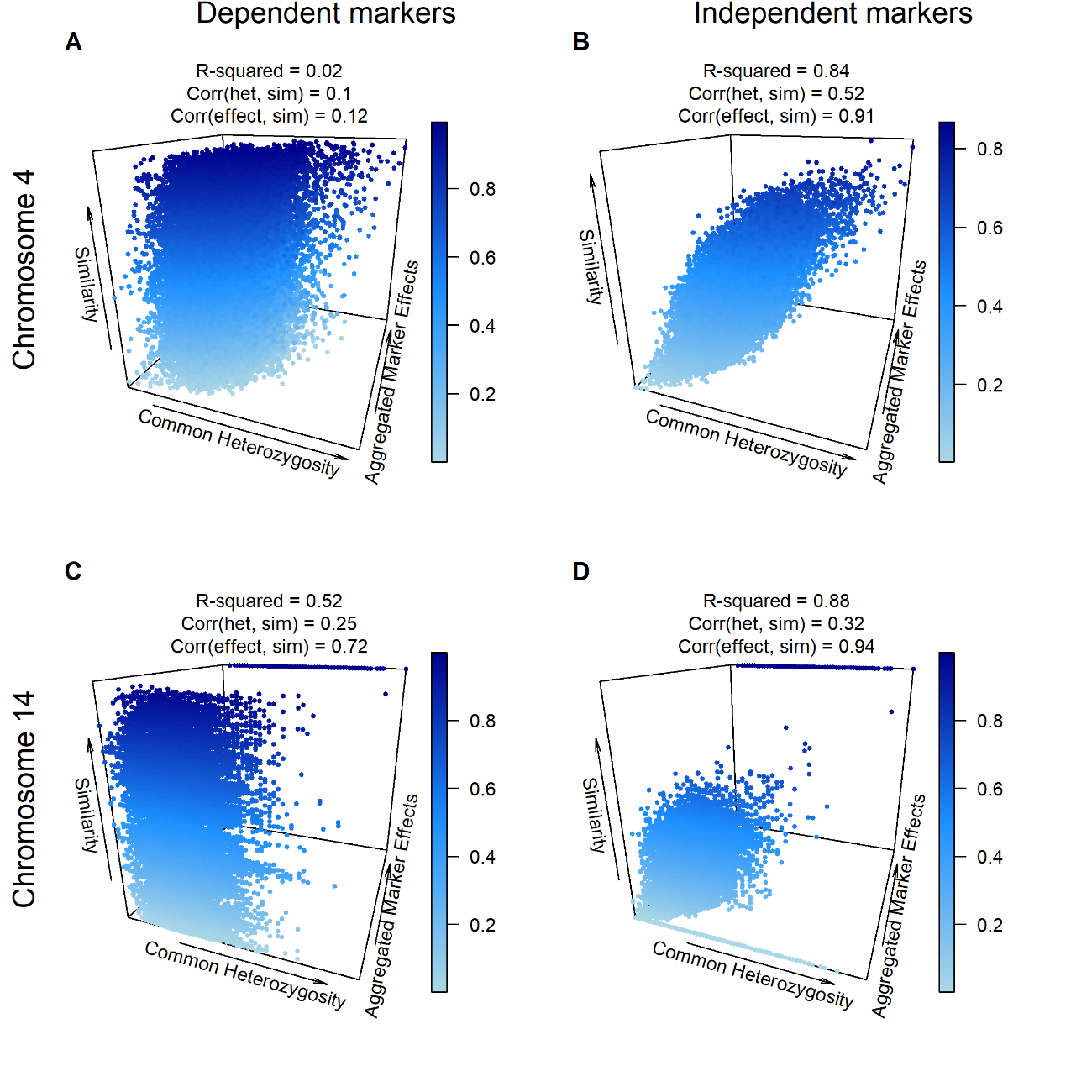


**Figure S9** Relationship between standardized haplotype similarities, common marker heterozygosity, and marker effect sizes in milk fat. Panels A and C depict dependent marker scenarios on chromosomes 4 and 14, respectively. Conversely, Panels B and D present independent marker scenarios on the same chromosomes, illustrating notable contrasts in relationships.


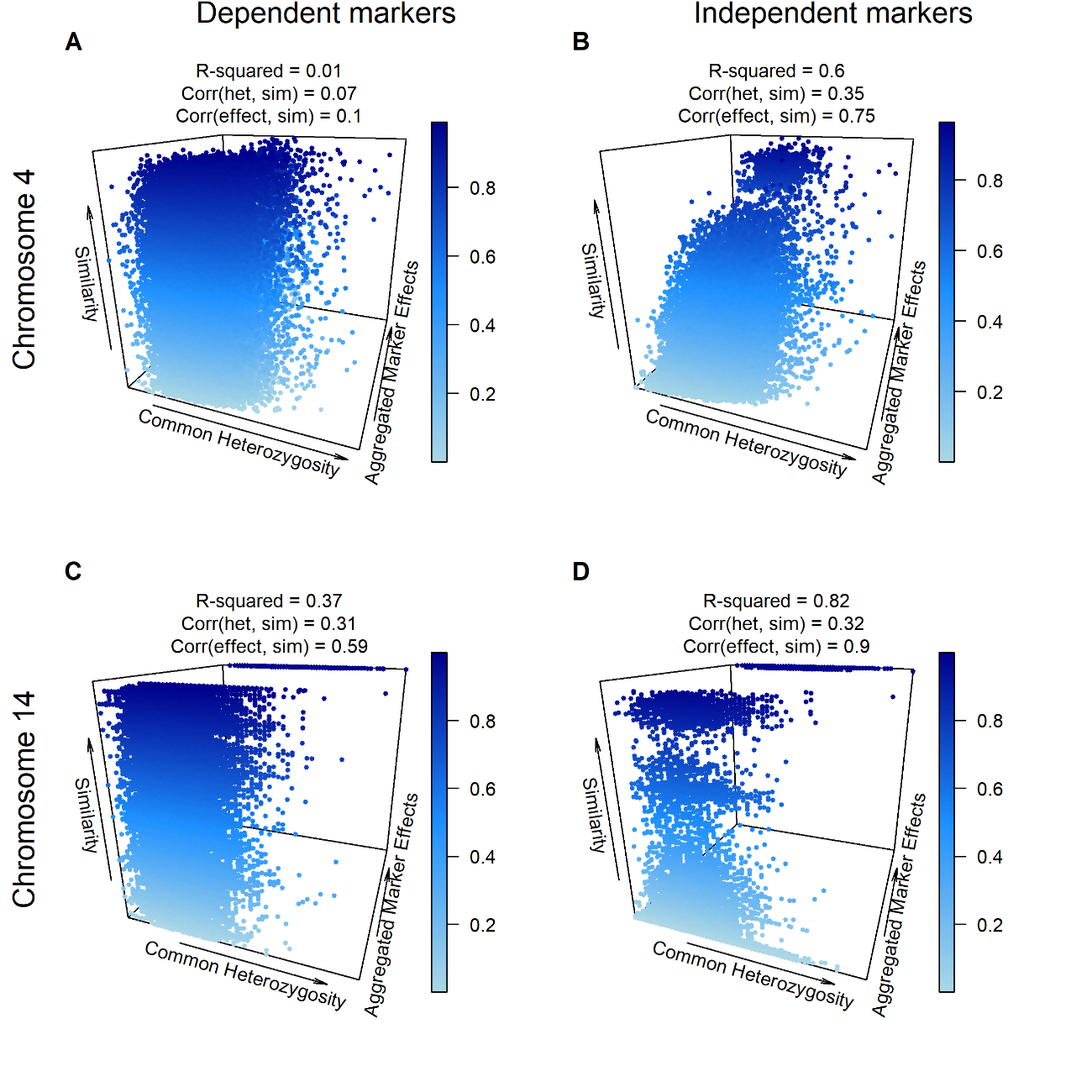


**Figure S10** Relationship between standardized haplotype similarities, common marker heterozygosity, and marker effect sizes in milk protein. Panels A and C depict dependent marker scenarios on chromosomes 4 and 14, respectively. Conversely, Panels B and D present independent marker scenarios on the same chromosomes, illustrating notable contrasts in relationships.


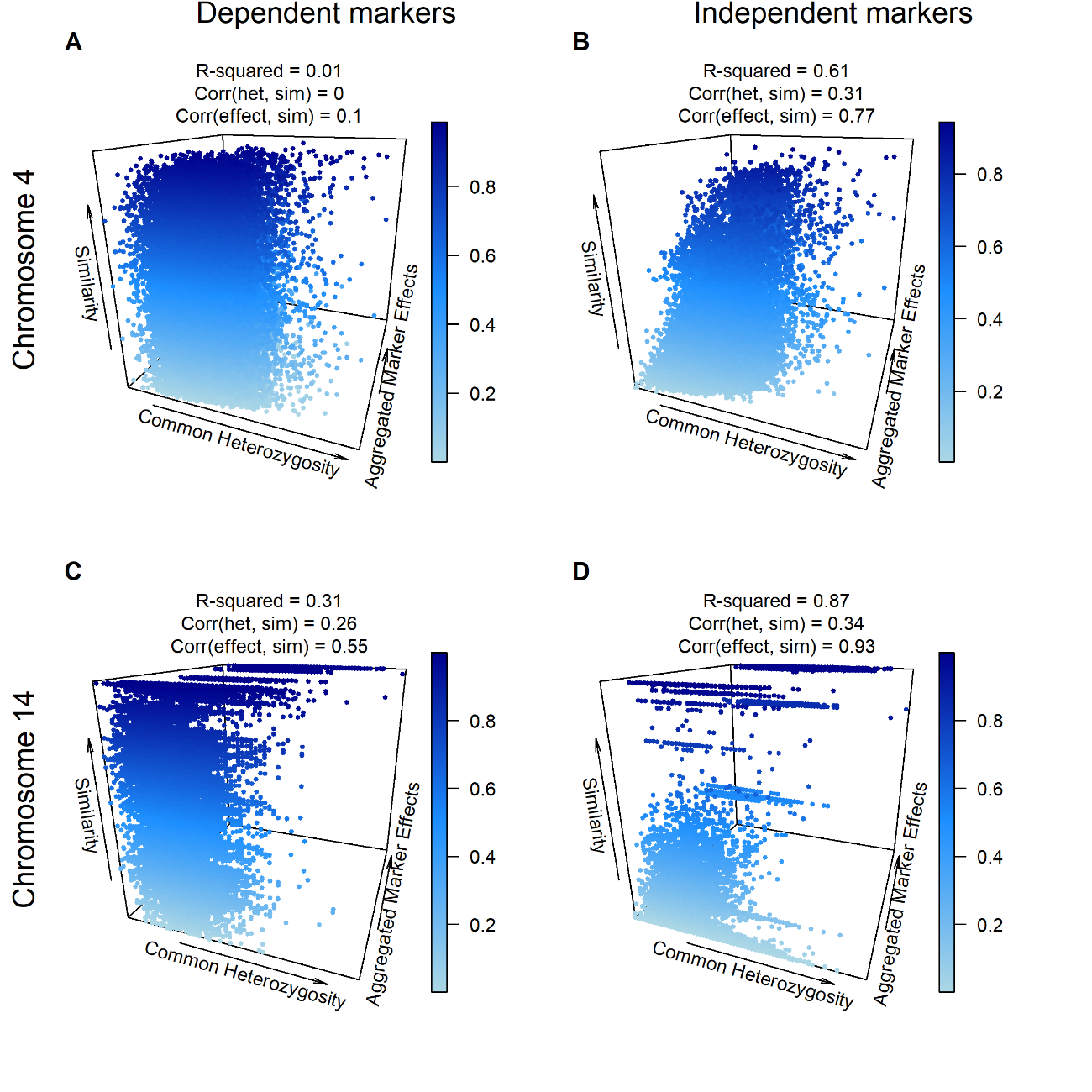


**Figure S11** Relationship between standardized haplotype similarities, common marker heterozygosity, and marker effect sizes in milk pH. Panels A and C depict dependent marker scenarios on chromosomes 4 and 14, respectively. Conversely, Panels B and D present independent marker scenarios on the same chromosomes, illustrating notable contrasts in relationships.


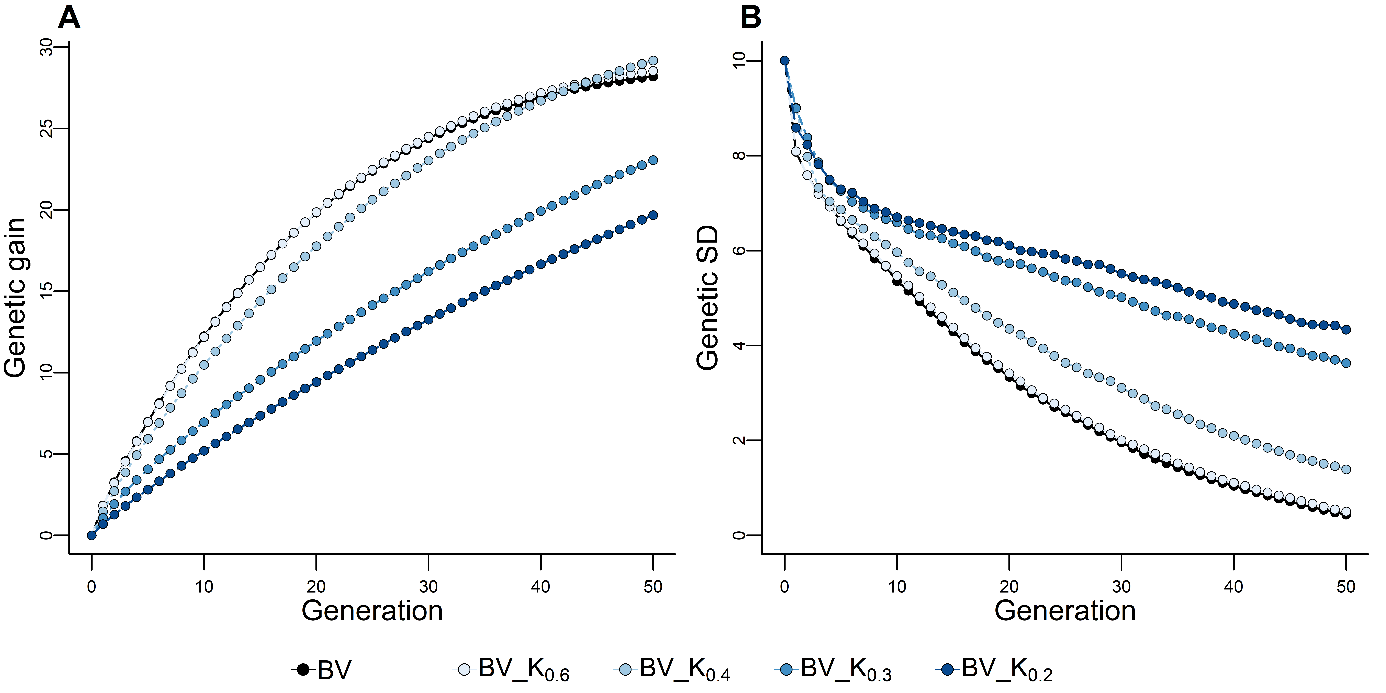


**Figure S12** Effect of similarity standardized matrix (K) on the cumulative genetic gain in genetic standard deviation (A) and genetic standard deviation (B). The selection schemes BV_K_0.6_(_0.4_, _0.3, 0.2_) optimize mate contribution by maximizing breeding value under various constraints (0.6, 0.4, 0.3, and 0.2) on the standardized haplotype similarity of parents. The results maximizing the index combining breeding value and Mendelian sampling variance are presented in Figure 6. Results are reported for 100 simulation runs.


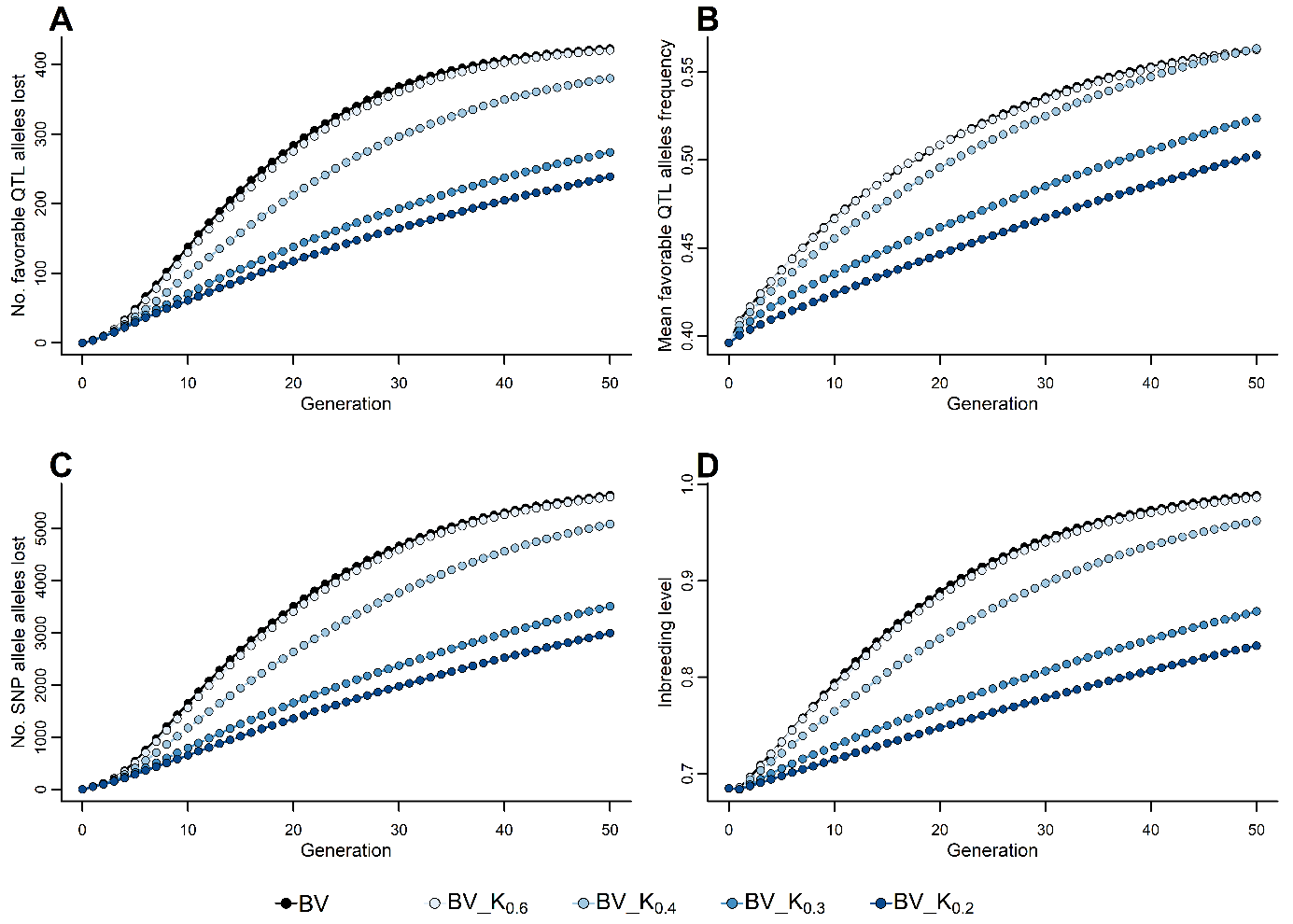


**Figure S13** Effect of standardized similarity matrix on favorable QTL alleles lost (A), mean favorable QTL allele frequency (B), SNPs lost (C), and expected inbreeding rate (D). The selection schemes BV_K_0.6_(_0.4_,_0.3,0.2_) optimize mate contribution by maximizing the breeding value (BV) under various constraints (0.6, 0.4, 0.3 and 0.2) on the standardized haplotype similarity of parents. The results maximizing the index combining breeding value and Mendelian sampling variance is presented in Figure 5. Results are reported for 100 simulation runs.


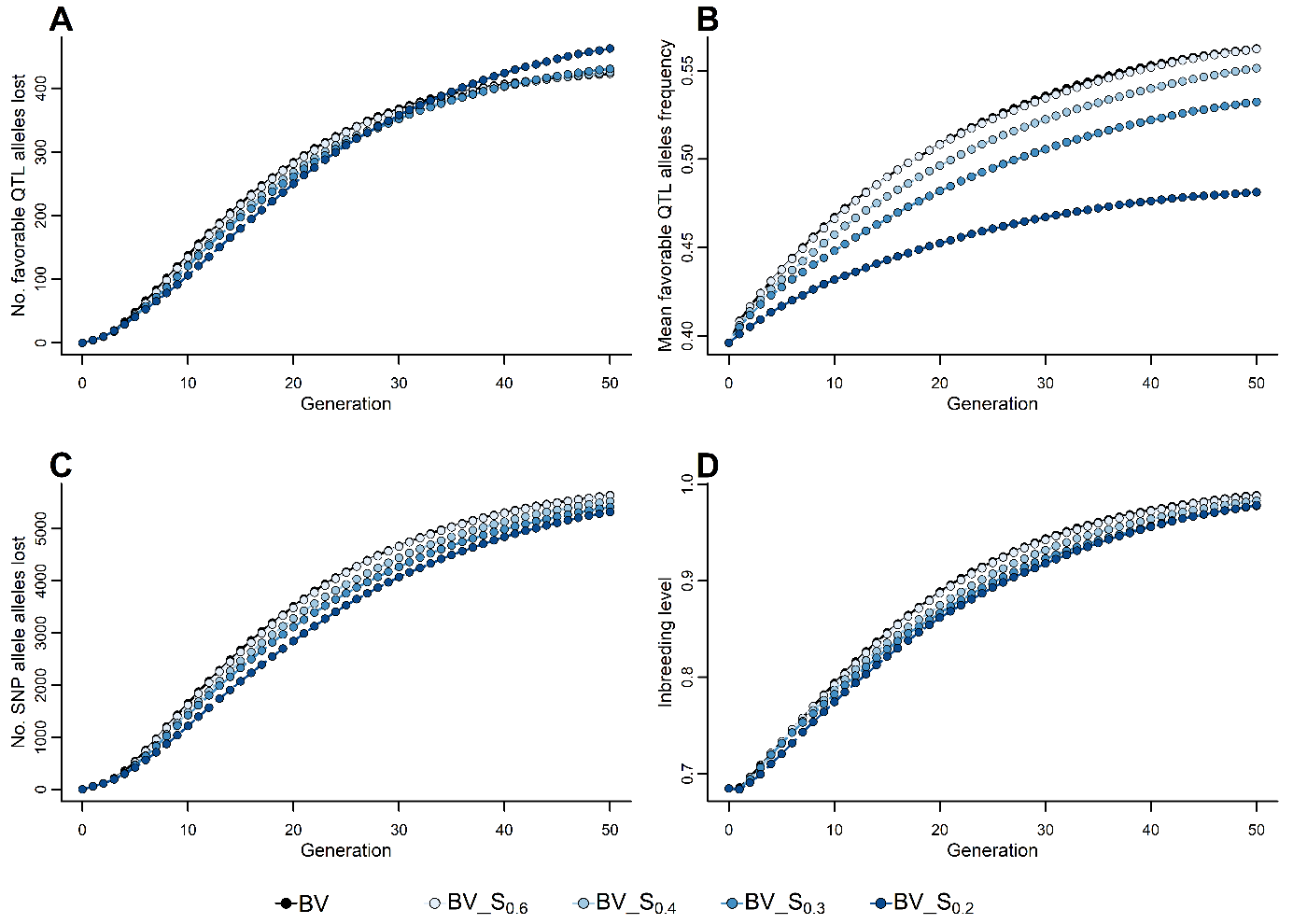


**Figure S14** Effect of similarity matrix on favorable QTL alleles lost (A), mean favorable QTL allele frequency (B), SNPs lost (C), and expected inbreeding rate (D). The selection schemes BV_S_0.6_(_0.4_,_0.3,0.2_) optimize mate contribution by maximizing the breeding value under various constraints (0.6, 0.4, 0.3 and 0.2) on the haplotype similarity of parents. Results are reported for 100 simulation runs.


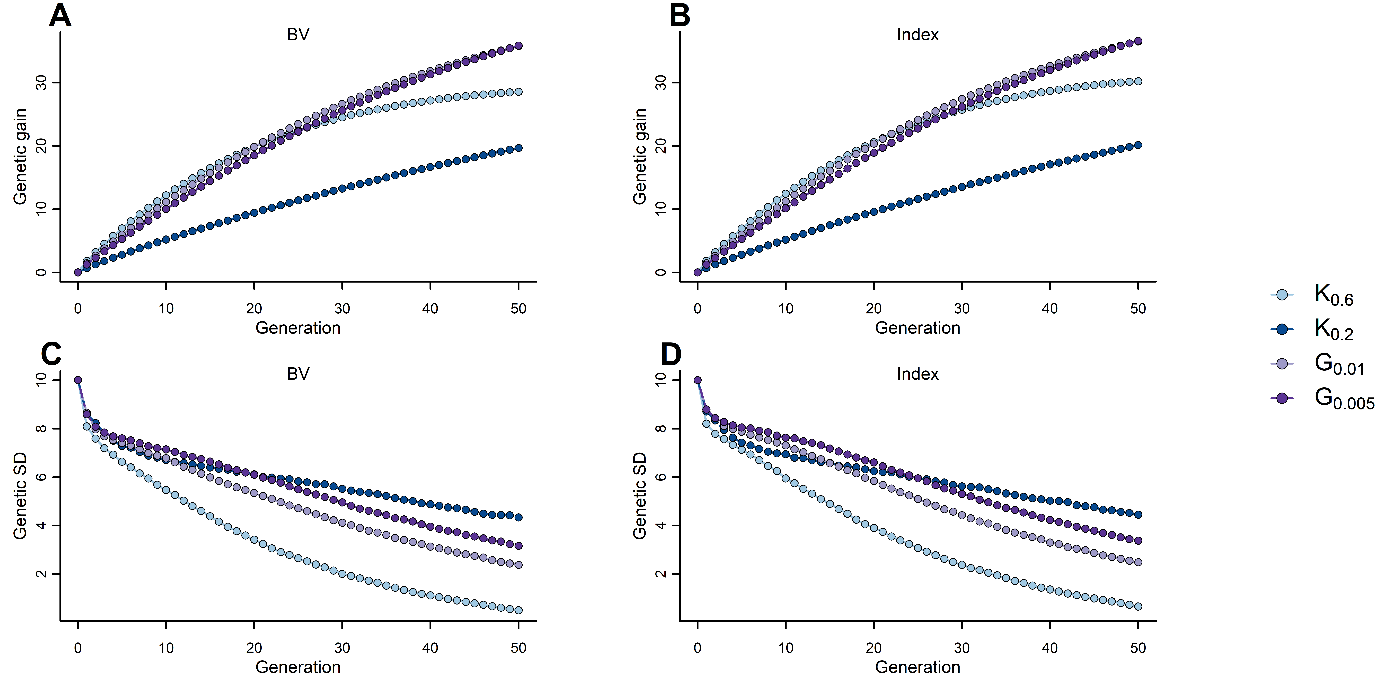


**Figure S15** Effect of standardized similarity matrix (K) and genomic relationship matrix (G) or their combination on the cumulative genetic gain in genetic standard deviation (A) and genetic standard deviation (B) under constraints to select at least five males. The selection schemes optimize mate contribution by maximizing breeding value (left panels) or index (right panels) within the constraints specified. In the case of K, the constraints are 0.6 and 0.2 for standardized haplotype similarity, and 1% or 5% inbreeding rate for G. Results are reported for 100 simulation runs.


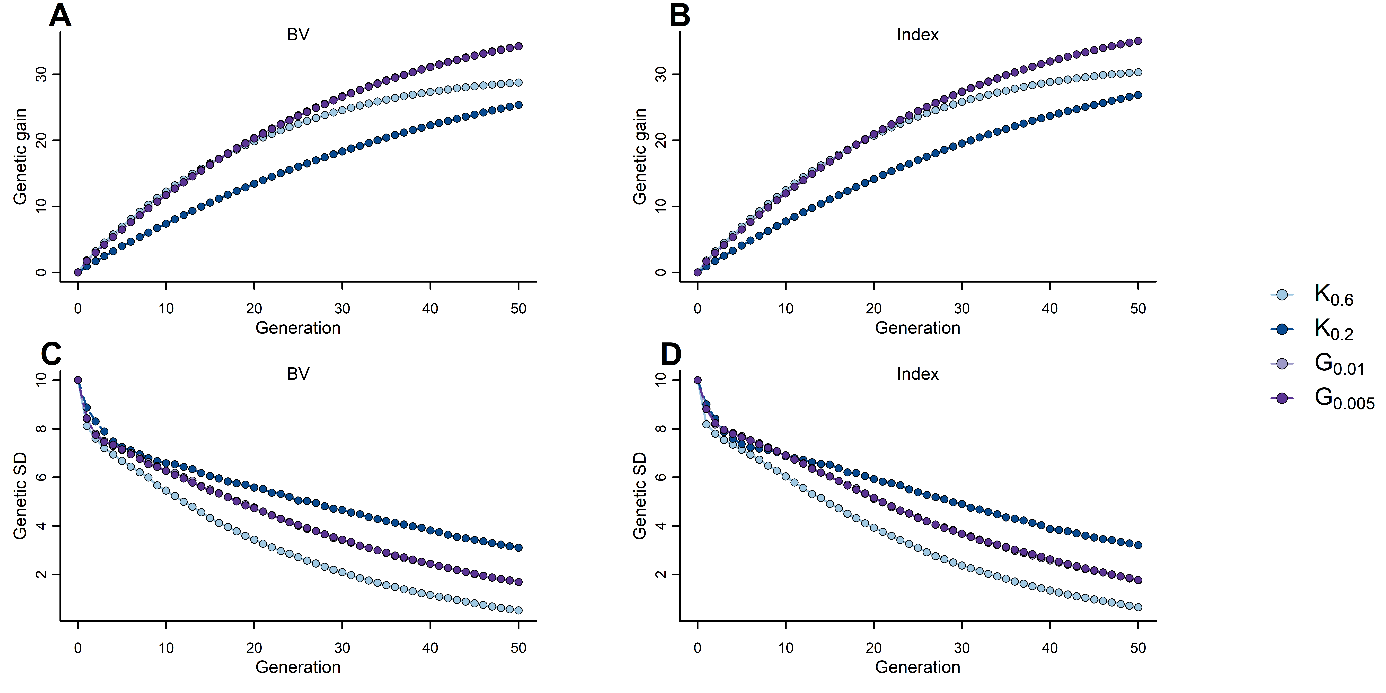


**Figure S16** Effect of standardized similarity matrix (K) and genomic relationship matrix (G) or their combination on the cumulative genetic gain in genetic standard deviation (A) and genetic standard deviation (B) under constraints to select at least 5 males and a maximum of 25 males. The selection schemes optimize mate contribution by maximizing breeding value (left panels) or index (right panels) within the constraints specified. In the case of K, the constraints are 0.6 and 0.2 for standardized haplotype similarity, and 1% or 5% inbreeding rate for G. Results are reported for 100 simulation runs.


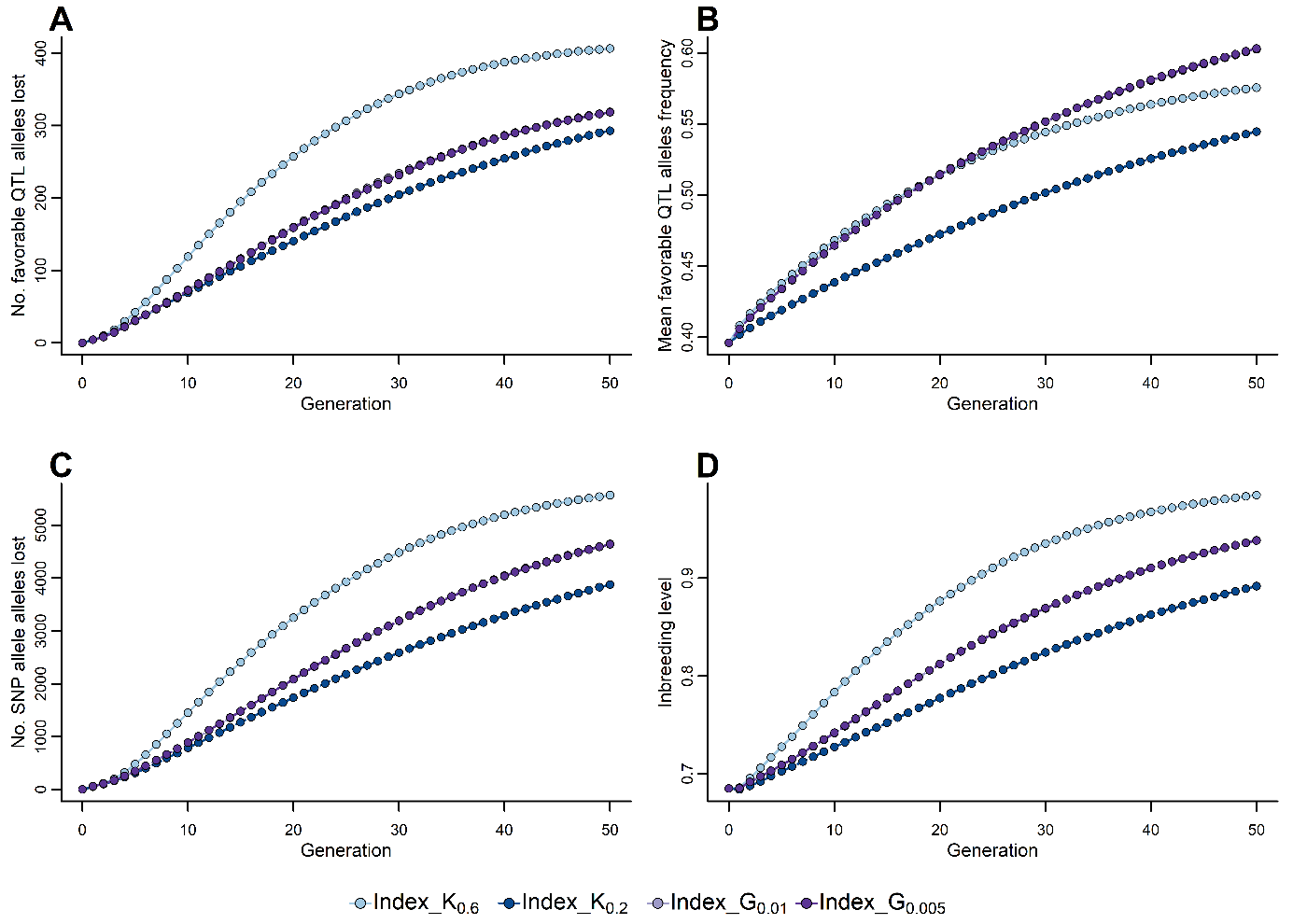


**Figure S17** Effect of standardized similarity matrix (K) and genomic relationship matrix (G) on favorable QTL alleles lost (A), mean favorable QTL allele frequency (B), SNPs lost (C), and expected inbreeding rate (D). The selection schemes optimize mate contribution by maximizing index within the constraints specified. In the case of K, the constraints are 0.6 and 0.2 for standardized haplotype similarity, and 1% or 5% inbreeding rate for G. Results are reported for 100 simulation runs.


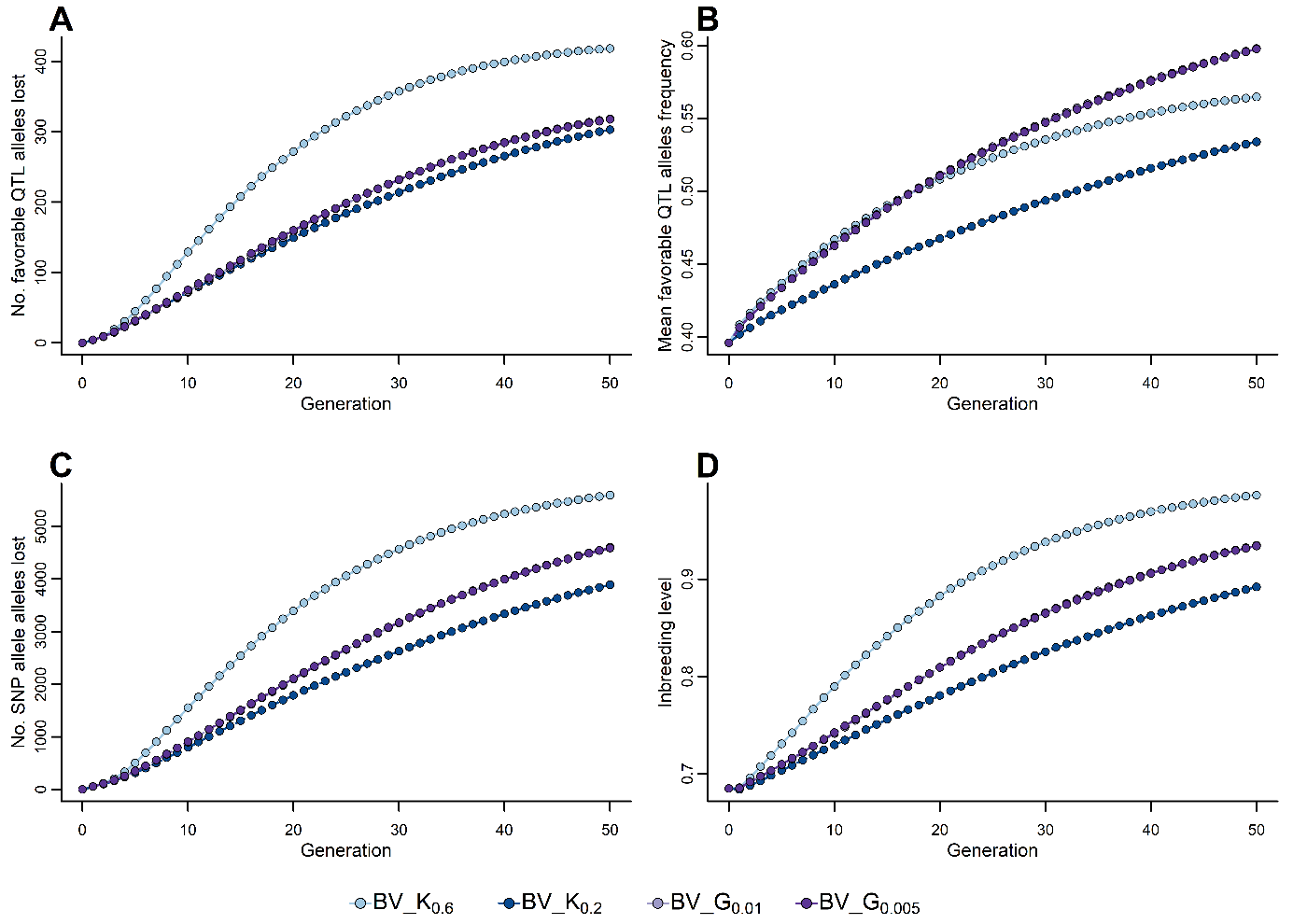


**Figure S18** Effect of standardized similarity matrix (K) and genomic relationship matrix (G) on favorable QTL alleles lost (A), mean favorable QTL allele frequency (B), SNPs lost (C), and expected inbreeding rate (D). The selection schemes optimize mate contribution by maximizing breeding value within the constraints specified. In the case of K, the constraints are 0.6 and 0.2 for standardized haplotype similarity, and 1% or 5% inbreeding rate for G. Results are reported for 100 simulation runs.
