# Supplementary material for: A Similarity Matrix for Preserving Haplotype Diversity Amongst Parents in Genomic Selection
Source: J Anim Breed Genet. 2025 Mar 4;142(6):652–68. doi: 10.1111/jbg.12930 (PMC12501756; doi:10.1111/jbg.12930)
Supplement: Supplementary file 5 — Data S5. [file JBG-142-652-s002.docx]

**SUPPLEMENTARY TABLES**

**Table S1:** Bivariate probability distribution of segregation patterns of gametes in parents $i$ and $j$

**Table S2:** Segregation patterns, probabilities of occurrence, marker haplotypes, and genetic values of zygotes produced by two pairs of parents ($ij$ and $uv$) with matching recombination patterns

**Table S3:** Summary Statistics for the number of selected males by various selection schemes

**Table S4:** Summary Statistics for the number of selected males by various selection schemes

**Table S5:** Summary statistics and properties of similarity matrices across generations

**SUPPLEMENTARY FIGURES**

**Figure S1:** A probability tree showing the segregation patterns (sequence of alleles from the first ($A$) and second ($B$) parental haplotypes), probabilities of occurrence, and the genetic values for trait $t1$ of the first ($g_{1}$) and second ($g_{2}$) parents.

**Figure S2:** A probability tree showing the segregation patterns (sequence of alleles from first ($A$) and second ($B$) parental haplotypes), probabilities of occurrence, and the genetic values for multiple traits ($t1$ and $t2$) of the first parent ($g_{1}$).

**Figure S3:** A probability tree showing the segregation patterns (sequence of alleles from first ($A$) and second ($B$) parental haplotypes), probabilities of occurrence, and the genetic values of zygotes from parent-pairs $ij$ and $uv$.

**Figure S4:** Similarity matrices showing chromosomes 4 and 14 for three milk fat, protein, and pH.

The red blocks demarcate each paternal half-sib family. Parents are arranged according to their pedigree.

**Figure S5:** Standardized similarity matrices showing chromosomes 4 and 14 for three milk fat, protein, and pH.

The red blocks demarcate each paternal half-sib family. Parents are arranged according to their pedigree.

**Figure S6:** Relationship between haplotype similarities, common marker heterozygosity, and marker effect sizes in milk protein.

Panels A and C depict dependent marker scenarios on chromosomes 4 and 14, respectively. Conversely, Panels B and D present independent marker scenarios on the same chromosomes, illustrating notable contrasts in relationships.

**Figure S7:** Relationship between haplotype similarities, common marker heterozygosity, and marker effect sizes in milk pH.

Panels A and C depict dependent marker scenarios on chromosomes 4 and 14, respectively. Conversely, Panels B and D present independent marker scenarios on the same chromosomes, illustrating notable contrasts in relationships.

**Figure S8:** Relationship between standardized haplotype similarities, common marker heterozygosity, and marker effect sizes in milk fat.

Panels A and C depict dependent marker scenarios on chromosomes 4 and 14, respectively. Conversely, Panels B and D present independent marker scenarios on the same chromosomes, illustrating notable contrasts in relationships.

**Figure S9:** Relationship between standardized haplotype similarities, common marker heterozygosity, and marker effect sizes in milk protein.

Panels A and C depict dependent marker scenarios on chromosomes 4 and 14, respectively. Conversely, Panels B and D present independent marker scenarios on the same chromosomes, illustrating notable contrasts in relationships.

**Figure S10:** Relationship between standardized haplotype similarities, common marker heterozygosity, and marker effect sizes in milk pH.

Panels A and C depict dependent marker scenarios on chromosomes 4 and 14, respectively. Conversely, Panels B and D present independent marker scenarios on the same chromosomes, illustrating notable contrasts in relationships.

**Figure S11:** Effect of similarity standardized matrix (K) on the cumulative genetic gain in genetic standard deviation (A) and genetic standard deviation (B).

The selection schemes BV_K_0.6_(_0.4_, _0.3, 0.2_) optimize mate allocation by maximizing breeding value under various constraints (0.6, 0.4, 0.3, and 0.2) on the standardized haplotype similarity of parents. The results maximizing the index combining breeding value and Mendelian sampling variance are presented in Figure 6. Results are reported for 100 simulation runs.

**Figure S12:** Effect of standardized similarity matrix on favorable QTL alleles lost (A), mean favorable QTL allele frequency (B), SNPs lost (C), and expected inbreeding rate (D).

The selection schemes BV_K_0.6_(_0.4_,_0.3,0.2_) optimize mate allocation by maximizing the breeding value (BV) under various constraints (0.6, 0.4,0.3 and 0.2) on the standardized haplotype similarity of parents. The results maximizing the index combining breeding value and Mendelian sampling variance is presented in Figure 5. Results are reported for 100 simulation runs.

**Figure S13:** Effect of similarity matrix on favorable QTL alleles lost (A), mean favorable QTL allele frequency (B), SNPs lost (C), and expected inbreeding rate (D).

The selection schemes BV_S_0.6_(_0.4_,_0.3,0.2_) optimize mate allocation by maximizing the breeding value under various constraints (0.6, 0.4, 0.3 and 0.2) on the haplotype similarity of parents. Results are reported for 100 simulation runs.

**Figure S14:** Effect of standardized similarity matrix (K) and genomic relationship matrix (G) or their combination on the cumulative genetic gain in genetic standard deviation (A) and genetic standard deviation (B) under constraints to select at least five males.

The selection schemes optimize mate allocation by maximizing breeding value (left panels) or index (right panels) within the constraints specified. In the case of K, the constraints are 0.6. and 0.2 for standardized haplotype similarity, and 1% or 5% inbreeding rate for G. Results are reported for 100 simulation runs.

**Figure S15:** Effect of standardized similarity matrix (K) and genomic relationship matrix (G) or their combination on the cumulative genetic gain in genetic standard deviation (A) and genetic standard deviation (B) under constraints to select at least 5 males and a maximum of 25 males.

The selection schemes optimize mate allocation by maximizing breeding value (left panels) or index (right panels) within the constraints specified. In the case of K, the constraints are 0.6. and 0.2 for standardized haplotype similarity, and 1% or 5% inbreeding rate for G. Results are reported for 100 simulation runs.

**Figure S16:** Effect of standardized similarity matrix (K) and genomic relationship matrix (G) on favorable QTL alleles lost (A), mean favorable QTL allele frequency (B), SNPs lost (C), and expected inbreeding rate (D).

The selection schemes optimize mate allocation by maximizing index within the constraints specified. In the case of K, the constraints are 0.6. and 0.2 for standardized haplotype similarity, and 1% or 5% inbreeding rate for G. Results are reported for 100 simulation runs.

**Figure S17:** Effect of standardized similarity matrix (K) and genomic relationship matrix (G) on favorable QTL alleles lost (A), mean favorable QTL allele frequency (B), SNPs lost (C), and expected inbreeding rate (D).

The selection schemes optimize mate allocation by maximizing breeding value within the constraints specified. In the case of K, the constraints are 0.6. and 0.2 for standardized haplotype similarity, and 1% or 5% inbreeding rate for G. Results are reported for 100 simulation runs.
